# SNHG9 promotes Hepatoblastoma Tumorigenesis via miR-23a-5p/Wnt3a Axis

**DOI:** 10.7150/jca.60748

**Published:** 2021-08-22

**Authors:** Sun Gui Feng, Rajeev Bhandari, Liu Ya, Bian Zhixuan, Pan Qiuhui, Zhu Jiabei, Mao sewi, Zhen Ni, Wang Jing, Sun Fenyong, Ma Ji, Ramesh Bhandari

**Affiliations:** 1 Department of Clinical Laboratory Medicine, Chengdu Second Peoples Hospital, Chengdu, Sichuan 610021, PR China.; 2 Department of Clinical Laboratory Medicine, Shanghai Tenth People's Hospital, Tongji University of Medicine Shanghai, China.; 3 Department of Clinical Laboratory Medicine, Shanghai Children's Medical Center, School of Medicine, Shanghai Jiaotong University, Shanghai, 200127, China.

**Keywords:** Hepatoblastoma, LncRNA, miRNA, miR-23a-5p, SNHG9

## Abstract

**Background:** Hepatoblastoma is a common hepatic tumor occurring in children between 0-5 years. Accumulating studies have shown lncRNA's potential role in distinct cancer progression and development, including hepatoblastoma. SnoRNA host gene 9 (SNHG9) is associated with the progression of distinct human cancers, but, its specific molecular mechanisms in hepatoblastoma is not unknown.

**Methods:** In this study, we estimated SNHG9 expression in hepatoblastoma tissue and cell lines by quantitative Real-Time Polymerase Chain Reaction (qRT-PCR). Next, we downregulated and upregulated SNHG9 expression in hepatoblastoma cell lines and then determined cell proliferation (CCK-8), colony formation, and cellular apoptosis activity. The dual luciferase reporter activity, RNA immunoprecipitation (RIP), biotin RNA pull down and Spemann's Pearson correlation coefficient assay were performed to establish the interaction between SNHG9, WNt3a and miR- 23a-5p. A xenograft *in-vivo* tumorgenicity test was performed to elucidate the role of SNHG9 hepatoblastoma in tumorigenesis. SNHG9 role in Cisplatin drug resistance in hepatoblastoma was also determined.

**Results:** SNHG9 was significantly upregulated in hepatoblastoma tissue and cell lines. SNHG9 overexpression on HUH6 & HepG2 resulted in a significant increase in cell proliferation and clonogenic activity while SNHG9 knock down resulted in a sustained inhibition of cell proliferation and clonogenic activity. Dual luciferase activity, RNA immunoprecipitation and biotin pull down confirmed the direct interaction of miR-23a-5p with SNHG9. The xenograft tumorgenicity test showed SNHG9 downregulation significantly inhibited the tumor growth in BALB/c mice. ROC and Kaplan-Meier analysis showed potential prognostic and diagnostic importance of SNHG9 in hepatoblastoma.

**Conclusion:** We concluded that SNHG9/miR-23a-5p/Wnt3a axis promotes the progression hepatoblastoma tumor.

## Introduction

Hepatoblastoma (HB) is the most commonly diagnosed primary embryonic hepatic tumor typically observed in children and toddlers between 0-5 years of age and rarely in adults 1, 2. It accounts for approximately 1% of all pediatric malignancies, meanwhile it accounts for only 65-90% of hepatic malignant tumors 3-6. The annual incidence rate of HB is estimated at 1.2-1.5 per million populations globally 7-10. The exact cause and pathophysiology of hepatoblastoma is mysterious and is believed to be originated from immature hepatic precursor cells. Increasing studies have shown their association with numerous genetic disorders, including Familial adenomatous polyposis (FAP), Beckwith-Wiedemann Syndrome, and Trisomy 18 11-14.

The WNT signaling cascade is an important key regulator of embryogenesis, organ development and homeostasis. However, abnormal Wnt signaling activation and deregulation have been linked to the onset, development and progression of distinct human malignancies and are the important hallmarks of cancer development 15, 16. A distinct wingless protein including Wnt1, Wnt2, Wnt3, Wnt3a, Wnt8b and others plays an important role in activation of Wnt signaling pathway activation and promotes the progression of distinct human cancers 17, 18. However, its role in hepatoblastoma is unknown. As a result, further research has to be carried out to understand the underlying uncovered molecular mechanisms of WNT signaling and Wnt3a in the aggressive development of hepatoblastoma tumors and to identify new therapeutic targets genes for the treatment of HB patients.

To date, treatment of HB patients mainly relies on surgical resection, neoadjuvant chemotherapy, and hepatic transplantation 19-23. Over the last few decades, a revolutionary change has occurred in diagnostic tools, surgical techniques, and chemotherapeutic treatment regimes, resulting in a substantial improvement in survival rates 24. However, there remains a small group (20-25%) of the HB patients with non-resectable HB tumors, chemoresistance and metastasis with a poor survival outcome. As a result, immediate action must be taken to identify and establish new molecular-genetic biomarkers, diagnostic and surgical techniques, and new chemotherapeutics target for the early diagnosis and effective treatment of HB patients.

Non-coding are the heterogeneous classes of RNA transcripts, including long noncoding RNA (lncRNAs), microRNAs (miRNAs) and others, which exert oncogenic and tumorgenicity functions 25, 26. Accumulating studies have shown that noncoding RNA, mainly lncRNAs (>200 nucleotides) and miRNAs (19-25 nt) have been aberrantly expressed in distinct human malignancies, and are being considered as an important emerging key player in the cancer paradigm. lncRNA influences the expression of miRNA targeted genes by interacting with miRNAs and reducing the regulatory effects of mRNAs 27, 28. For examples, LncRNA UCA1 sponges with miR-240-5p to promote glioma via upregulation of ZEB1. Similarly, in papillary thyroid carcinoma, LncRNA Gas5 controls the PTEN expression by sponging miR-222-3p 29, 30. Small nucleolar RNA host gene 9 (SNHG9) 551 nucleotide base pair intergenic lncRNA located on chr16p13.3 have all been linked to the onset and progression of distinct cancers including pancreatic cancer 21, glioblastoma 22, lung cancer 23, ovarian cancer 24. However, the exact function and underlying mechanism of SNHG9 in hepatoblastoma tumorigenesis is unknown and needs to be explored.

Similarly, increasing evidences has shown that deregulated miRNA is associated with cancer progression 31, 32. For instance, miR-21 promotes hepatocellular carcinoma 33, miR-34s is upregulated in hepatoblastoma 34, miR-675 is upregulated in osteosarcoma 35. Previous researchers have shown the dysregulation of miR-23a-5p in distinct cancers including pancreatic ductal adenocarcinoma (downregulation), glioblastoma and esophageal carcinoma 36-39.

Nevertheless, the function of miR-23a-5p in hepatoblastoma tumor progression is undiscovered and need to be explored.

In the current study, we investigated the expression level, specific functional and underlying molecular mechanisms of SNHG9 and miR-23a-5p. Our study findings, demonstrate that SNHG9 is significantly upregulated in hepatoblastoma tissue and is closely associated with poor prognosis in hepatoblastoma. SNHG9 promotes hepatoblastoma tumorigenesis through wnt3a/miR-23a-5p axis. Hence, SNHG9/miR-23a-5p/wnt3a might be a novel and promising therapeutic target for hepatoblastoma patients.

## Material and Methods

### Human clinical specimens

Between 2016 and 2020, a total of 40 pairs of hepatoblastoma tissue, including the adjacent matching non-tumorous hepatic tissues, were obtained from pediatric patients who underwent hepatic surgery at Shanghai Tenth Peoples Hospital, China. Participants of this study were selected based on the inclusion and exclusion criteria. The inclusion criteria for the participants in our study were: (1) The pediatric patients between 0-5 years, confirmed for hepatoblastoma tumors based on histopathological evaluation, and had never undergone radiotherapy, chemotherapy, and other adjuvant treatment prior to surgery. The exclusion criteria were: (1) Pediatric patients with a multiple cancer who underwent chemotherapy, or radiation therapy were excluded from the study. This study was approved by the Institutional Research Ethical Review Committee. Verbal and written consents were obtained from all the pediatric patient's parents prior to collection of human clinical samples for research.

### Hepatoblastoma cell line cultures

The ATCC Hepatoblastoma cell lines (HUH6 & HepG2) and normal hepatic cell lines (QSG7701) were obtained from the Shanghai Chinese Academy of Cell Collection. HUH6, HepG2 and QSG- 7701 cells were cultured and maintained in Dulbecco's Modified Eagles medium (DMEM), Minimum Essential Medium (MEM) and RPMI 1640 respectively, supplemented with 10% FBS and 100 U/ml Penicillin G/Streptomycin. The hepatoblastoma (HUH6 & HepG2) cells and normal hepatic (QSG7701) cells were maintained in a humidified 5% CO2 incubator at 37 °C. All the culture media (DMEM, MEM & RPMI) and FBS used in this research were purchased from Gibico (Grand Island, NY, USA).

### Hepatoblastoma (HUH6 and HepG2) cell transfections

For transient knockdown of the target gene (SNHG9) in hepatoblastoma cell lines transfection, three candidates for short interfering SNHG9 RNAs (si-SNHG9#1, si-SNHG9#2 and si-SNHG9#3) along with negative control (si-NC) were chemically synthesized by Gene Pharma (Shanghai). Meanwhile, for the stable silencing of SNHG9 in hepatoblastoma (HUH6 & HepG2), short hair pin RNAs (shRNAs: shSNHG9#1m shSNHG9#2 and shSNHG9#3) and SNHG9 overexpression plasmid were synthesized by Keli Biotechnology (Shanghai, China). In addition, miR23a-5p mimic, miR23a-5p inhibitor and matched negative controls were synthesized by Shanghai Gene Pharma Inc (Shanghai, China). Briefly, hepatoblastoma cell (HUH6 & HepG2) cells with a density of 2.5 × 105 cells/wells were seeded on a 6-well plate and incubated for 18-24 h at 37 °C to allow confluency growth of 30-40%. After 24 h of cell growth on 6 well plates, the hepatoblastoma cell lines (HUH6 & HepG2) were transfected with SNHG9siRNAs/SNHG9shRNAs/miR-23a-5p and their negative controls using Lipofectamine 2000 according to manufactured guidelines. At 48 h of post transfection, HB cells were harvested for qRT-PCR or western blot analysis. The sequences of SNHG9 siRNA, hsa-miR-23a-5p/mimics/inhibitors are enlisted in **Supplementary File 1.**

### RNA Extraction and Quantitative real-time PCR (qRT-PCR)

Total mRNA from the hepatoblastoma primary tissues and cell lines was extracted using the Trizol regent (Invitrogen), as per the manufacturer's protocols. The total mRNA was reversed transcribed into cDNA using the PrimeScript RT reagent kit (Takara, Dalian, P.R China). Meanwhile, microRNAs were reverse transcribed into cDNA using a miRNA-specific loop RT primer (Ribobio, Guangzhou, China). Amplification of target genes (SNHG9, Wnt3a c-MYC, β-catenin and miR23a-5p) was carried out using SYBR Premix Ex Taq II (Takara Biotechnology, China) on a ABI Prism 7500 machine (Applied Biosystems, Thermo scientific). GAPDH and U6 were used as as endogenous controls for mRNAs and miRNA respectively. The relative fold changes in mRNA/miRNAs expression were calculated by the 2^-∆∆CT^ method. The primers sequence for distinct of the target genes and miRNAs used in this study are enlisted in **Supplementary File 1.**

### Protein extraction and Western Blotting

The protein from the transfected hepatoblastoma (HUH6 & HepG2) cells was extracted using the RIPA lysis buffer (Beyotime, China) supplemented with a protease inhibitor (PI) cocktail (Cell Signaling Technology, USA) and phenylmethanesulfonylfluoride (PMSF) (Beyotime, China). The protein concentration in the protein lysate was determined using a BCA kit (Beyotime, China). Protein (80 µg) was separated by 10% SDS-PAGE and blotted (transferred) into the nitrocellulose membrane. The nitrocellulose membrane was blocked with 5% BSA blocking solution for 60 min. The nitrocellulose membrane was incubated with specific primary antibodies including Wnt3a (ab2194120, β-catenin (ab32572), β-Actin (ab170325) (1:5000), C-Myc (ab32072), Blc2 (ab182858), Bax (ab32503) overnight at 40c. Following the overnight incubation, the nitrocellulose membrane was rinsed four times with PBST and incubated with secondary, HRP- conjugated goat anti-rabbit antibody (ab6721) at room temperature for 90-120 mins in room temperature. Next, the nitrocellulose membrane was treated with enhanced chemiluminescence (ECL) chromogenic western blotting substrates to visualize the separated protein band on the nitrocellulose membrane using an Amersham^TM^ A600 chemiluminescence film scanner (GE Healthcare Life Sciences). ß-actin was used as an internal control for the validation of protein loading samples. The separated protein was quantified by densitometry (Image J Software).

### Cell proliferation test

The effect of SNHG9/miR-23a-5p silencing and overexpression in hepatoblastoma cells (HUH6 & HepG2) on cell proliferation activity was assessed by the CCK-8 assay (CCK-8, Beyotime, China). Briefly, transfected hepatoblastoma cell lines (HUH6 & HepG2) were seeded in 96 wells at a density of 1×10^3^ cells/well and incubated at humidified 5% CO2 incubator at 37 °C for 5 days. 10 µl of CCK8 solution (Beyotime, China) was added to each well and incubated for 2 h at 37 °C. Finally, the cell proliferation activity was determined every 24 h (0, 24, 48, 72, 96 & 120 h) using a BioTek multi-mode microplate reader (BioTek, USA) at 450 nm. Cell proliferation activity was normalized with zero hours' time absorbance.

### Clonogenic Assay

Transfected hepatoblastoma (HUH6 & HepG2) cells were seeded into a 6-well plate at a density of 1 × 10^3^ per wells and incubated in a humidified 5% CO2 incubator at 37 °C for 14 days and the culture medium of each well are replaced every 3 days. Briefly, following14 days of incubation, the culture medium from the 6-well plates was pipetted out and washed thrice with 1× PBS. This is followed by the fixation of the cell colonies with 4% paraformaldehyde and then stained with 0.05% crystal violet. Eventually, the crystal violet from the 6 well plates were removed and washed with tap water and allowed the plate to air dry. After air drying the images were taken and the number of colonies in each well was counted using the Image J software, and presented in a bar chart using the Graph prism.

### Flow Cytometric analysis for Apoptosis Assay

To determine the cellular apoptosis activity on transfected hepatoblastoma (HUH6 & HePG2) cells we utilized the Annexin V fluorescein isothiocyanate (FITC)/ propidium iodide double staining Apoptosis Detection Kit. In brief, following the trypsinization, the transfected HB cells were collected in tubes and centrifuged at 1000 RPM for 5 mins. Afterward, cells collected on the bottom of the tube were washed twice with ice cold 1× PBs and then suspended in Annexin binding buffer. The cell suspension was then distributed into distinct tubes at a density of 1×10^5^ cells/tube followed by the double staining solution. Initially, the cell suspension was stained with Annexin-V FITC for 15 minutes and then with propidium iodide (PI) for 5 minutes. Eventually, flow cytometry (BD Biosciences company, USA) was utilized to detect the apoptotic cells. The Flow Jo Software was used to calculate the percentage of cellular apoptosis.

### Subcellular Cytoplasmic and Nuclear Distribution of RNA

The Ambion PARIS Kits (Invitrogen, NY, USA) have been used for the isolation of cytoplasmic and nuclear fractional RNA in hepatoblastoma mammalian cell lines (HUH6 & HepG2) in accordance with a manufacture protocol, followed by qRT-PCR to estimate the relative fractional distribution of SNHG9, U6, and GAPDH in HB cell lines. U6, 18s and GAPDH were used as nuclear and cytoplasmic control transcripts.

### RNA Immunoprecipitation Assay (RIP) Assay

The EZMagnna RNA-binding protein immunoprecipitation kit (Millipore, MA, USA) was utilized to validate the interaction between SNHG9 and miR23a-5p and was performed in accordance with manufactured guidelines. In brief, pCDNA-SNHG9 or miR23a-5p mimics transfected HUH6 and HepG2 cells were plated on a 6 well plate and incubated for 48 h. After 48 h of transfection, HB cell lysate was obtained after the subsequent treatment of HUH6 and HepG2 cells with RIP lysis buffer. Magnetics beads coated with Ago2 antibody (Millipore's, USA) or anti rabbit IgG (Milipore's, USA) antibody mixed with cell lysate buffers and incubated for 6 h at 4 °C. After 6 h, immunoprecipitated RNA was extracted with the subsequent elution of protein beads. Eventually, qRT-PCR was performed to analyze the extracted precipitated RNA. Total RNA was considered as an input control.

### Biotin Pulldown Assay

Biotin label antisense and sense SNHG9 RNA and DNA probes were designed, synthesized and purchased from Sangoon Biotech (Shanghai, China). The hepatoblastoma (HUH6) cell lysate was mixed with the biotinylated SNHG9 RNA/ DNA probes and was incubated approximately at 25 °C for 1 h. The streptavidin-agarose beads (Invitrogen) were mixed into the mixture to elute the biotin- coupled RNA complexes. Eventually, qRT-PCR was performed to assess the abundance of SNHG9 and has-miR23a-5p in pull-down materials.

### Xenograft Tumors

*In-vivo* animal tumorigenicity experiment was performed to demonstrate the oncogenic potential of SNHG9. 4-week-old BALB/c nude mice of were used for this experiment. BALB/c nude mice were purchased and randomly classified into two major groups: sh-NC and sh-SNHG9 and each group consisted of six BLAB/C nude mice. For the tumorgenicity assay, sh-NC and sh-SNHG9 stably knockdown 5×10^6^ HUH6 cell suspension were injected subcutaneously into the posterior flank of each BALB/c nude mice. After 7 days of injection of HUH6 cell suspension into mice, the tumor growth, size and body weight of each mouse were measured in every three days until 24 days. The volume of the tumors was measured using the equation V=0.5 ×D ×d^2^, where V= volume, D is the longitudinal diameter and d is the latitudinal diameter of tumors. At the end of 21 days all the mice were killed by cervical dislocations. Thereafter, the weight of tumors and volume of tumor were measured. All animal experiments were carried out in compliance with the NIH guidelines for the care and use of laboratory animals.

### Bioinformatics analysis

Similarly, we utilized the RegRNA 2.0 database to identify the possible miRNA binding to SNHG9. Similarly, we utilized the RNA hybrid database to determine the possible binding site of SNHG9 to miR-23a-5p. Similarly, we utilized the Target scan database to identify the miR-23a- 5p targeting genes. Similarly, we utilized Starbase and RNAhybrid to identify the binding sites of miR-23a-5p in the Wnt3a target gene.

### Dual Luciferase Assay

The 3'-UTR putative target binding site of SNHG9 on miR-23a-5p and miR-23a-5p binding sites on Wnt3a were synthesized and cloned into pmirGLO (PsiCheck2) firefly luciferase vectors (Promega, Madison, WI, USA). Briefly, HUH6 and HepG2 cells were seeded at the density of 1×10^5^ cells in a 12 well plate and incubated at 37 °C for 24 h. SNHG9 (SNHG9-WT & SNHG9- Mut) and Wnt3a (Wnt3a-WT & Wnt3a-Mut) constructed vectors were co-transfected into HepG2 and HUH6 cells with miR23a-5p mimics/inhibitors using Lipofectamine 2000 (Invitrogen, USA). After 48 h of co-transfection, the HepG2 and HUH6 cells were lysed using passive lysis buffer and collected. Eventually, relative luciferase activity on cell lysate was determined using the Dual-Luciferase Reporter Assay system (Promega, China).

### Statistical analysis

All the findings of this study were presented in mean±SD. Statistical analysis was conducted using SPSS version 16.0 (IBM, NY, USA) and GraphPad prism version 8.0 (GraphPad Software, La Jolla, CA). The Student's t-test and one-way ANOVA were used to calculate and evaluate the two and more groups. The Spearman's rank correlation coefficient test was used to determine the correlation between two groups. Study findings with p value ≤ 0.05 were defined as statistical significance.

## Results

### SNHG9 is upregulated in hepatoblastoma tumor tissues and cell lines

**Figure 1:**
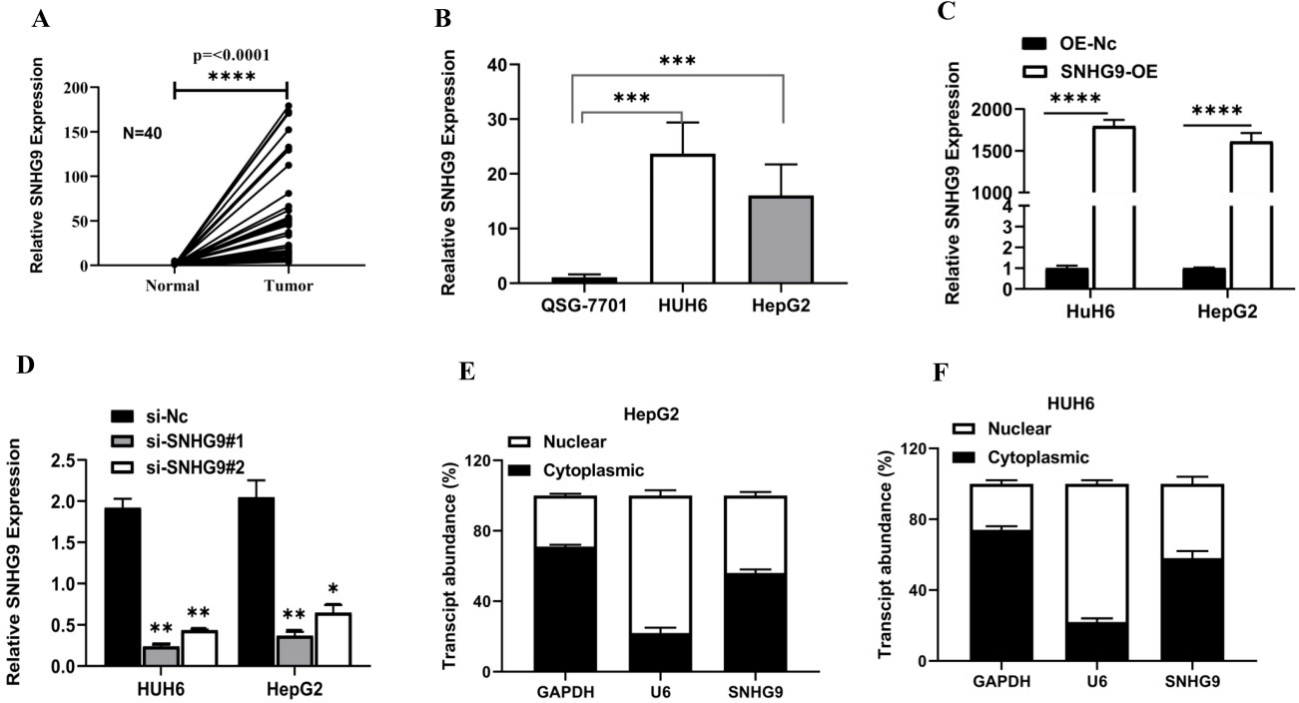
** SNHG9 is upregulated in hepatoblastoma tumor tissues and cell lines. (A-B)** SNHG9 mRNA expression in hepatoblastoma tissue and cell lines (HUH6 & HepG2) was determined by qRT-PCR. **(C)** SNHG9 expression in SNHG9 overexpression plasmid transfected HepG2 and HUH6 was determined by q-RT-PCR. **(D)** Relative expression of in SNHG9 knockdown HHU6 and HepG2 cells. **(E-F)** Fractional distribution of SNHG9, GAPDH and U6 in HepG2 and HUH6 estimated by qRT-PCR. All the data is shown as the mean ±SD. *p-value<0.05, **p<0.01, ****p<0.0001.

SNHG9 was found to be significantly upregulated in primary hepatoblastoma tissue compared to adjacent normal hepatic tissue (**Figure 1A**). Further, we examined the relative SNHG9 expression in hepatoblastoma (HUH6 & HepG2) cell lines and found a remarkable high expression of SNHG9 in hepatoblastoma (HUH6 & HepG2) cell lines than in QSG7701 normal hepatic cell lines (QSG7701) (**Figure 1B**). Next, we investigated the effect of SNHG9 knockdown and overexpression on the relative expression of SNHG9 in HUH6 and HepG2 cells. Among the SNHG9 upregulated HUH6 & HepG2, there was significantly high expression of SNHG9 (**Figure 1C**). Conversely, a significant reduction in SNHG9 expression was observed among SNHG9 knockdown/downregulated hepatoblastoma (HUH6 & HepG2) cell lines (**Figure 1D**). Next, we observed the subcellular distribution of SNHG9 in hepatoblastoma (HUH6 & HepG2) cells and revealed that SNHG9 was predominately located in the cytoplasm like that of the majority lncRNA (**Figure 1E and 1F**). Based on the above findings, we confirmed that SNHG9 is highly overexpressed in the hepatoblastoma tissue and cell lines, suggesting that it may promote hepatoblastoma tumorigenesis.

### SNHG9 expression correlation with clinicopathological factors and SNHG9 upregulation and its prognostic and diagnostic value in Hepatoblastoma

**Figure 2:**
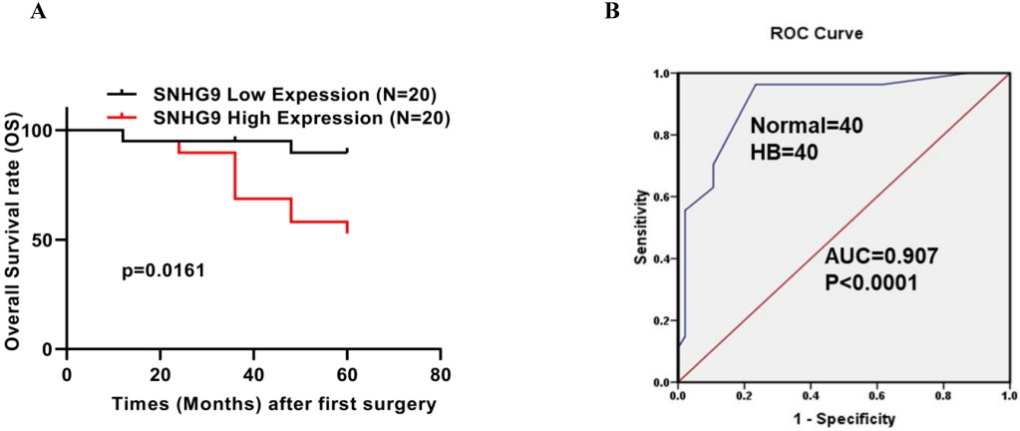
** Prognostic and diagnostic significance of SNHG9. (A)** The Kaplan-Meier analysis demonstrated, an overall survival rate (OS) rate among hepatoblastoma patients with low and high SNHG9 expression. **(B)** ROC curved analysis showed SNHG9 has a high degree of sensitivity and specificity in the diagnosis of HB patients, making it a valuable diagnostic tool. All the data's are shown as the mean ± SD. **p<0.01, ****p<0.0001.

SNHG9 expression among hepatoblastoma tumor patients was significantly upregulated (**Figure 1A**). Next, to better understand the significance of SNHG9 upregulation in hepatoblastoma, we performed a correlation test to elucidate the association between SNHG9 expression and patients' clinicopathological characteristics. SNHG9 expression was significantly correlated with histology types of HB tumor (p=0.056). However, no significant correlation was found between the SNHG9 expression and other clinical features such as age, gender, tumor size, and TNM staging (**Table 1**). Next, we performed the Kaplan-Meier survival analysis to determine the overall survival (OS) rate among 40 HB patients (20 high SNHG9 expression and 20 low SNHG9 expression patients) and found hepatoblastoma patients with high SNHG9 expression had a low 5-year survival rate (p=0.0161) compared to hepatoblastoma patients with low SNHG9 expression (**Figure 2A**). In addition, we performed a ROC curve analysis to determine the diagnostic significance value of SNHG9 and found that it has a high degree of sensitivity and specificity (AUC=0.8928; p-value<0.0001) (**Figure 2B**). Taking into consideration of the above findings, SNHG9 may serve as an independent predictor of overall survival. And it could be a potential promising novel biomarker for the prompt early diagnosis and prognosis of HB patients.

### SNHG9 silencing and overexpression impacts on Hepatoblastoma cell lines cellular proliferation, colonization, and apoptosis activity

**Figure 3:**
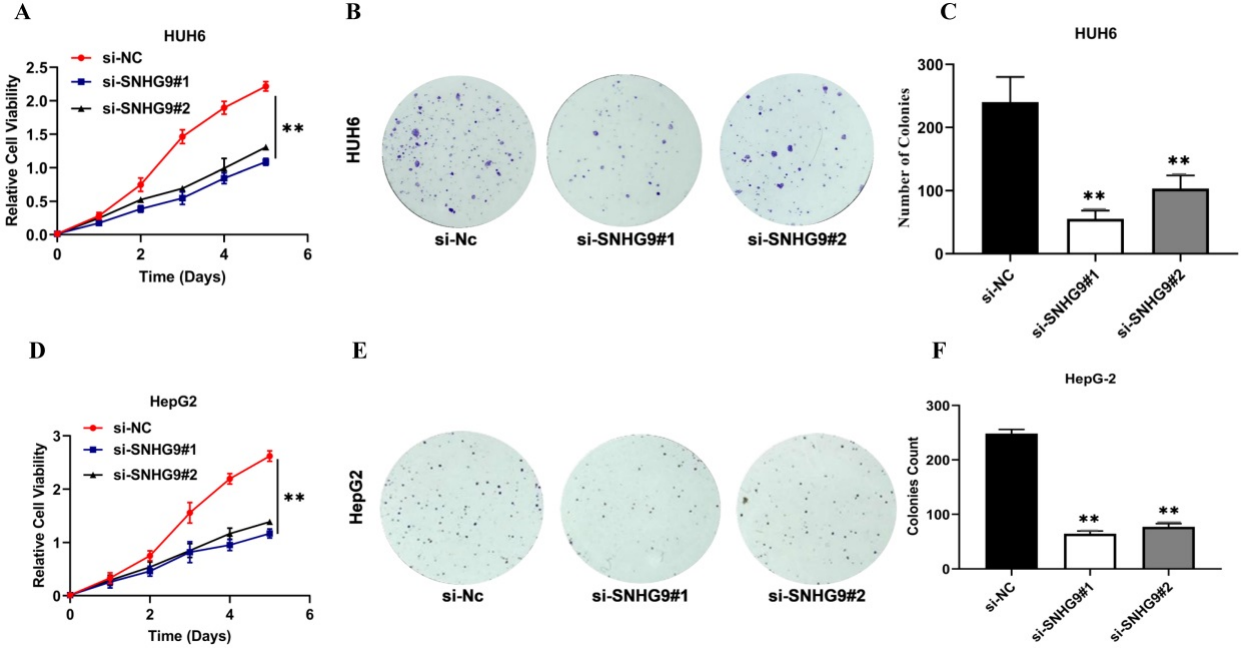

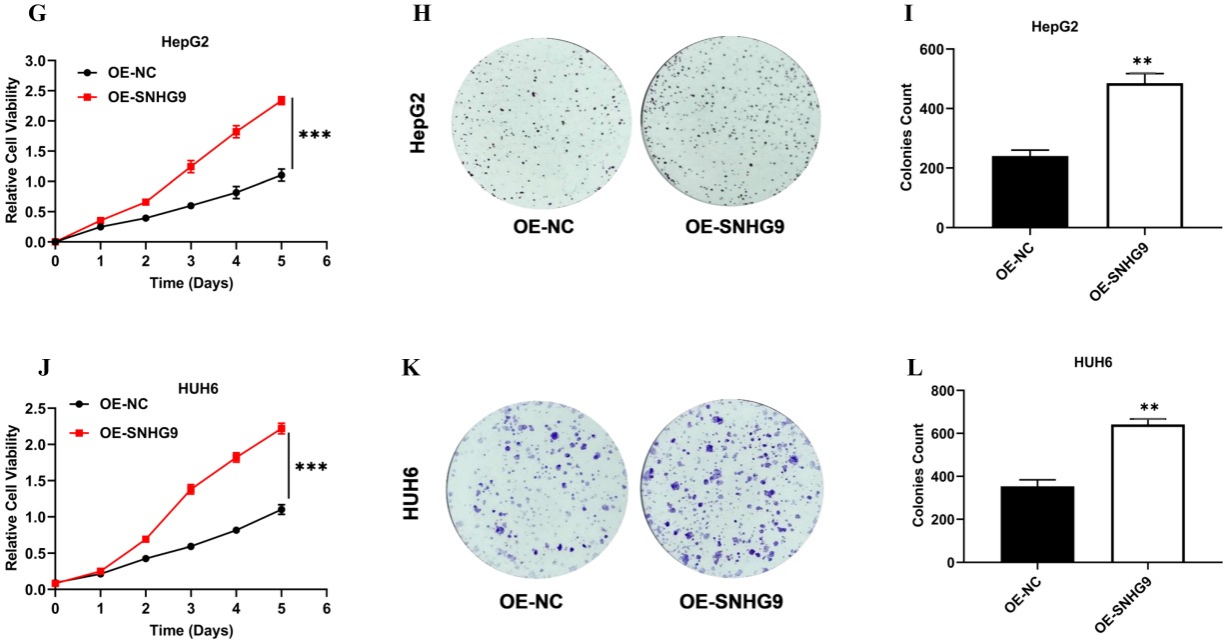

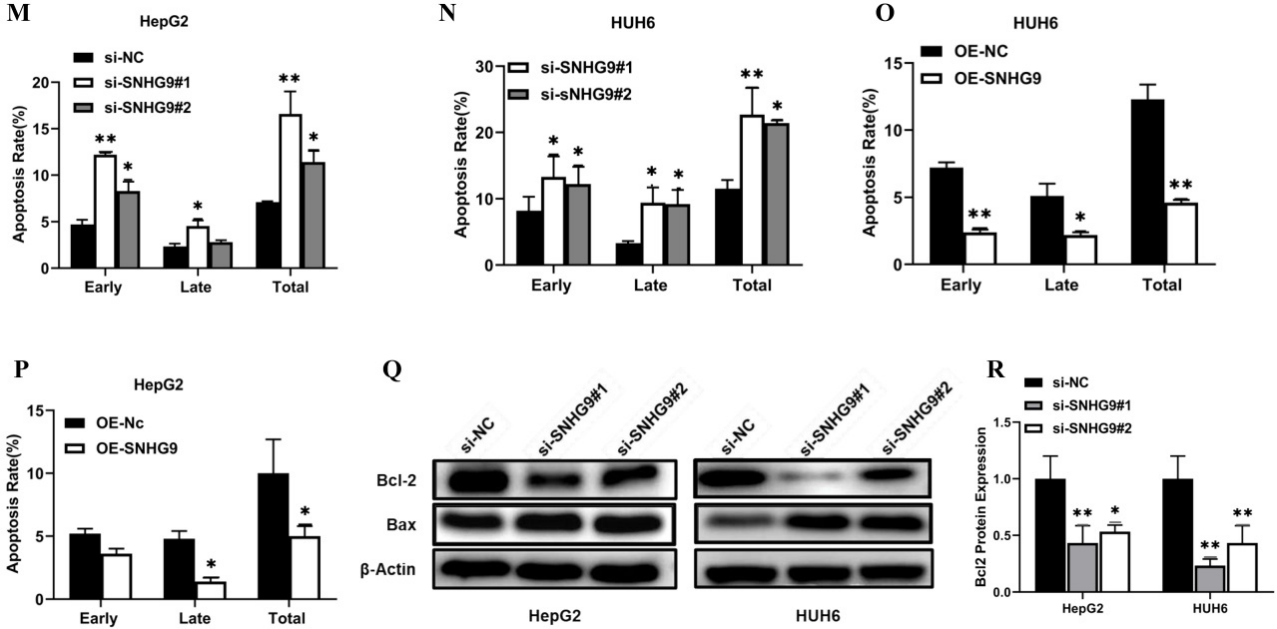

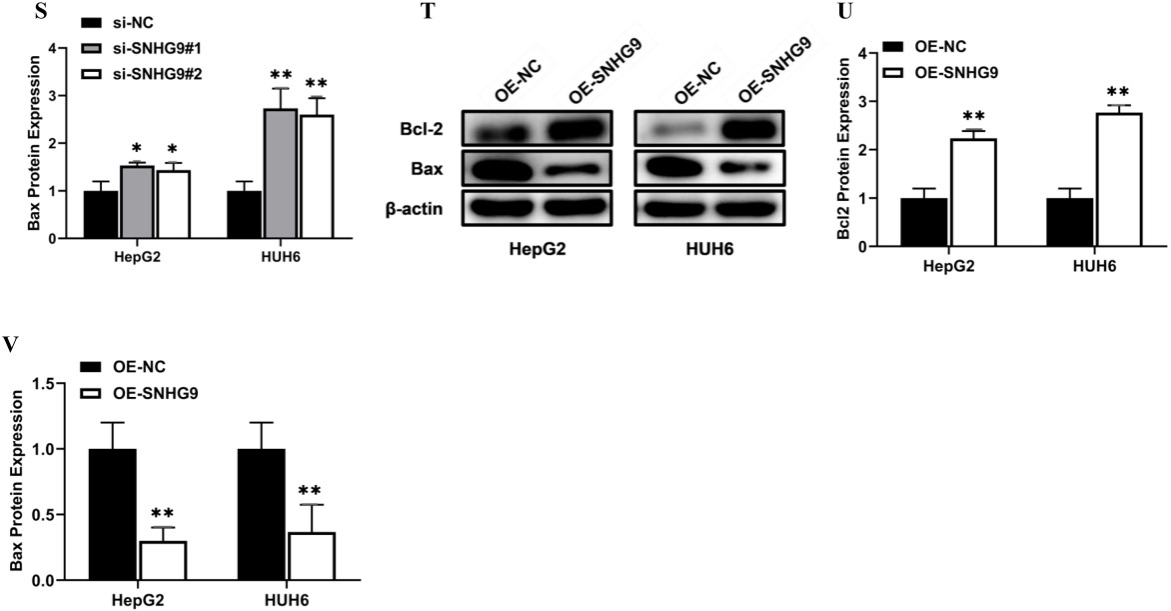
** (A-H) SNHG9 silencing and overexpression impact on hepatoblastoma cell proliferation, clonogenic and apoptosis activities. (A-F)** A decreased in cell proliferation and clonogenic activity was reported in SNHG9 knock down in HepG2 and HuH6 cells. **(G-L)** Increased cellular proliferation and clonogenic activity was in SNHG9 overexpressed HUH6 and HepG2 cell. **(M-N)** Increased cellular apoptosis activity reported in SNHG9 knockdown HUH6 and HepG2 cell and was determined by flow cytometry. **(O-P)** Decreased cellular apoptosis activity in SNHG9 overexpressed HUH6 and HepG2 cells. **(Q-S)** A decreased in anti-apoptotic (Bcl-2) protein and an increased in apoptosis protein (Bax) was observed in SNHG9 knockdown HepG2 and HUH6 cells. **(T-V)** Increased Bcl-2 anti-apoptotic and decreased Bax apoptosis protein expression were reported in SNHG9 overexpressed HUH6 and HepG2 cells. All the data are shown as the mean ± SD. *p-value<0.05, **p<0.001, ***p<0.0001.

The effect of SNHG9 silencing and overexpression on hepatoblastoma (HUH6 and HepG2) cell proliferation, colony formation, and cellular apoptotic activity were investigated (**Figure 3A-V**). The functional silencing of SNHG9 in HUH6 and HepG2 cells results in significant decrease in cell proliferation (CCK8) and clonogenic activity (**Figure 3A-F**); conversely, SNHG9 overexpression in HB (HUH6 & HepG2) cells shows a remarkable increase in their cell proliferative and clonogenic activity (**Figure 3G-L**).

Furthermore, we investigated the effect of SNHG9 silencing and overexpression on hepatoblastoma cellular apoptosis activity. Flow cytometry analysis findings, showed that SNHG9 silencing efficiently enhances cellular apoptosis in HUH6 & HepG2 cells (**Figure 3M and 3N**).

Conversely, SNHG9 overexpression results in significant depletion of cellular apoptosis activity in HUH6 & HepG2 cells (**Figure 3O and 3P**).

Furthermore, we performed a western blot analysis to demonstrate the consequences of SNHG9 silencing and overexpression on the expression level of Blc2 and Bax proteins. SNHG9 silencing results in a considerable increase in Bax protein levels and a remarkable reduction in Bcl2 protein expression (**Figure 3Q and 3S**). Conversely, SNHG9 overexpression in HepG2 and HUH6 results in a remarkable increase in Bcl2 protein and a significant reduction in Bax protein (**Figure 3T-V**). Based on the above findings, we established that SNHG9 enhances cellular proliferation and supports the growth and progression of HB tumors.

### SNHG9 downregulates miR-23a-5p activity

Initially, we investigated the fractional subcellular localization of SNHG9 in HUH6 and HepG2 cells. Subcellular localization assay results showed that higher proportions of SNHG9 located in the cytoplasm and to a lesser extent in the nucleus of HepG2 and HUH6 cells (**Figure 1E and 1F**). Accumulating evidences has shown that most cytoplasmic lncRNAs act as competing endogenous RNA (ceRNA) or molecular sponges for specific miRNAs, thus influencing their biological activity. Next, we identified the target for SNHG9 using the RegRNA database and identified 4miR-23a-5p as the target miRNAs.

**Figure 4:**
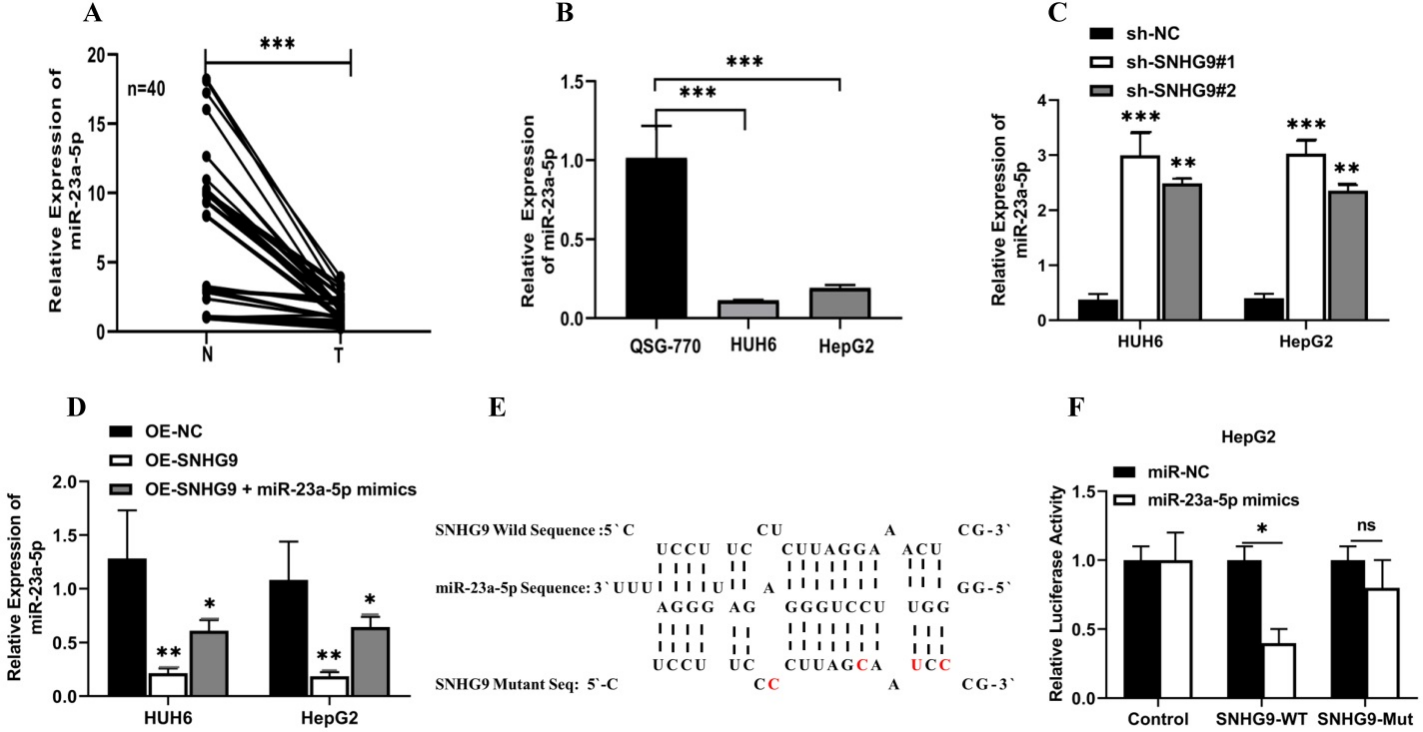

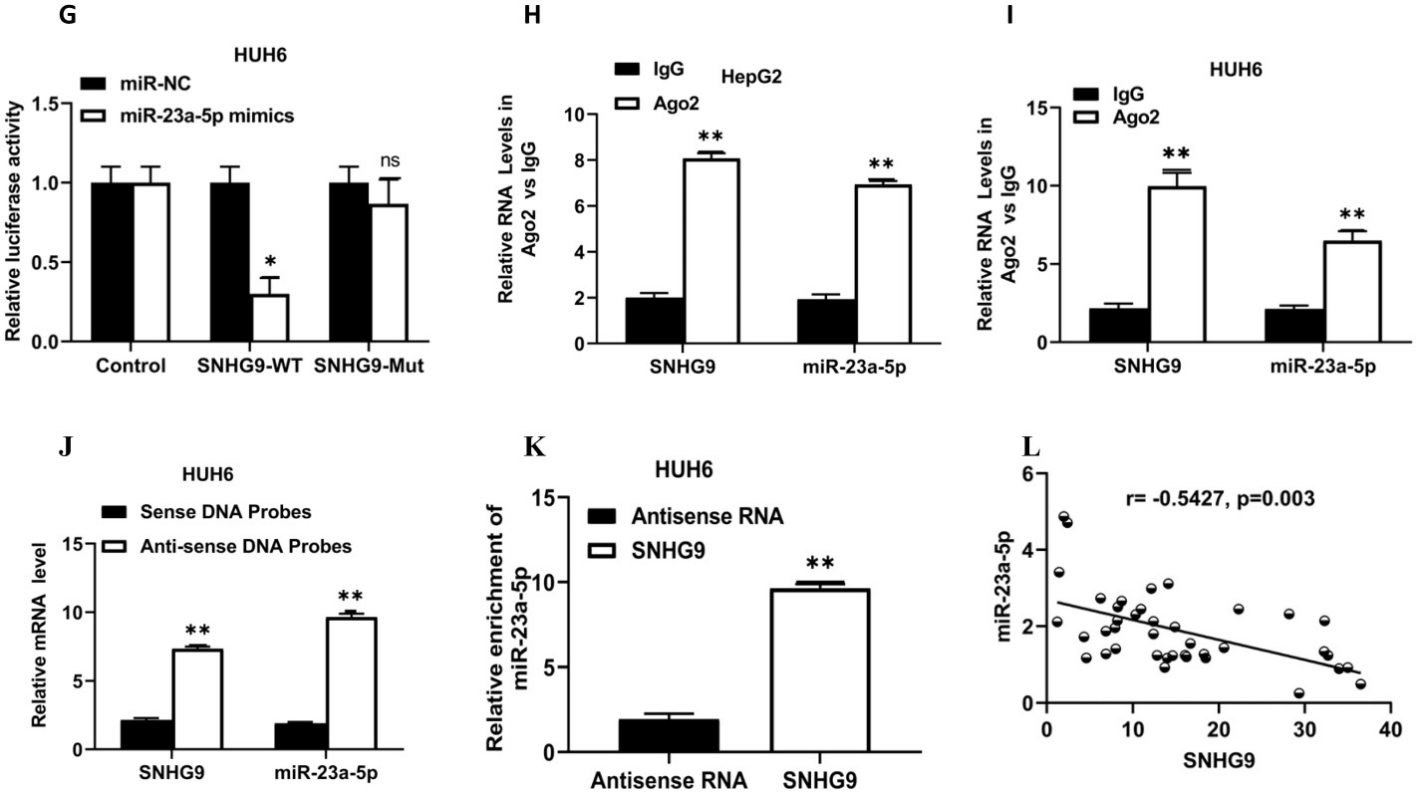
** SNHG9 downregulates miR-23a-5p expression. (A-B)** miR-23a-5p expression in primary hepatoblastoma tissue and hepatoblastoma cell lines determined by qRT-PCR. **(C)** miR-23a-5p expression in SNHG9 downregulated HUH6 and HepG2 determined by qRT-PCR. **(D)** Relative expression of miR-23a-5p in SNHG9 upregulated HUH6 and HepG2 cells. **(E)** The predictive binding sites of bindings sites SNHG9 in miR-23a-5p. **(F-K)** Dual luciferase assay, RIP and biotin RNA Pull down assay to assess the interaction between SNHG9 and miR-23a-5p. **(L)** Spearman correlation analysis to explore the correlation in between SNHG9 and miR-23a-5p. All data are presented as the mean ± SD. *p-value<0.05, **p<0.01, ***p<0.001 ****p<0.0001.

Furthermore, we investigated the expression of miR-23a-5p in hepatoblastoma tissue and cell lines and found a significant lower expression of miR-23a-5p in primary hepatoblastoma tissue than in normal hepatic tissue and QSG7701 normal hepatic cell lines (**Figure 4A and 4B**). Next, we investigated the effect of SNHG9 downregulation and overexpression of SNHG9 on the relative expression of miR-23a-5p. The qRT-PCR analysis result showed a significantly high expression of miR-23a-5p was seen in SNHG9 knockdown HUH6 and HepG2 cells (**Figure 4C**). Meanwhile, a significant reduction in miR-23a-5p expression was reported in SNHG9 overexpressed HUH6 & HepG2 cells (**Figure 4D**).

Further, using the online bioinformatics RNA hybrid software, we identified the predictive putative 3'-UTR binding sites of SNHG9 in miR23a-5p (**Figure 4E**). Next, we performed a dual luciferase assay to demonstrate the interaction in between SNHG9 and miR-23a-5p. A considerable low luciferase activity was observed in HuH6 and HepG2 cells co-transfected with SNHG9-WT (Wild) reporter vector and miR-23a-5p mimics, suggesting that SNHG9 interacts with miR-23a-5p and interferes with the biological activity of miRNA (**Figure 4F and 4G**).

Likewise, a RIP assay was performed on HUH6 and HepG2 cell extracts using antibodies against Argonauted (Ago2) antibodies and normal IgG antibodies and found a significant enrichment of SNHG9 and miR-23a-5p in Ago2 immunoprecipitated RNA relative to control IgG immunoprecipitated RNA (NC control) (**Figure 4H and 4I**), suggesting a strong interaction between miR23a-5p and SNHG9. Similarly, Biotin pulldown has also been used to confirm the interaction between SNHG9 and miR23a-5p. The biotin pull down assay showed high enrichment of SNHG9 and miR-23a-5p in anti-sense DNA probes than in sense DNA probes (**Figure 4J and 4K**). Furthermore, Spearman's correlation analysis demonstrated association between relative expression of SNHG9 and miR23a-5p, and found that SNHG9 expression was negatively correlated with miR23a-5p expression (**Figure 4L**). All of these experimental findings indicate that SNHG9 interacts directly to miR-23a-5p and negatively modulates its functions.

### miR-23a-5p downregulation promotes hepatoblastoma tumorigenesis

**Figure 5:**
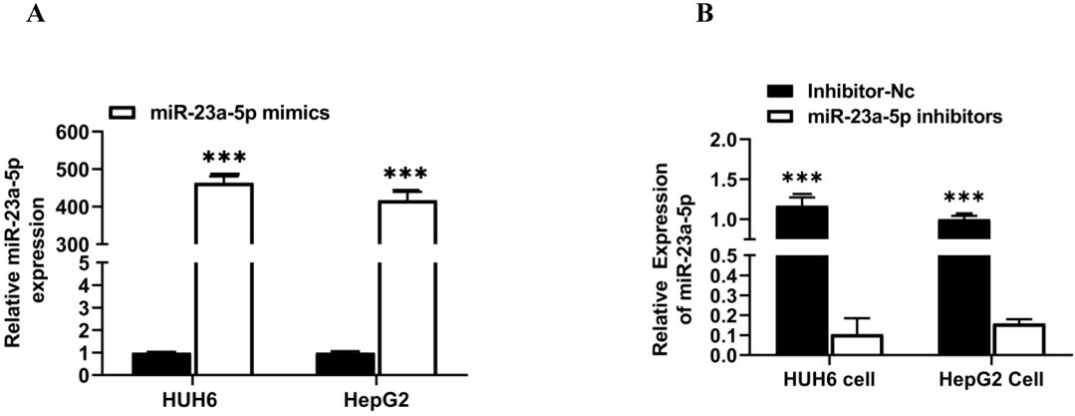

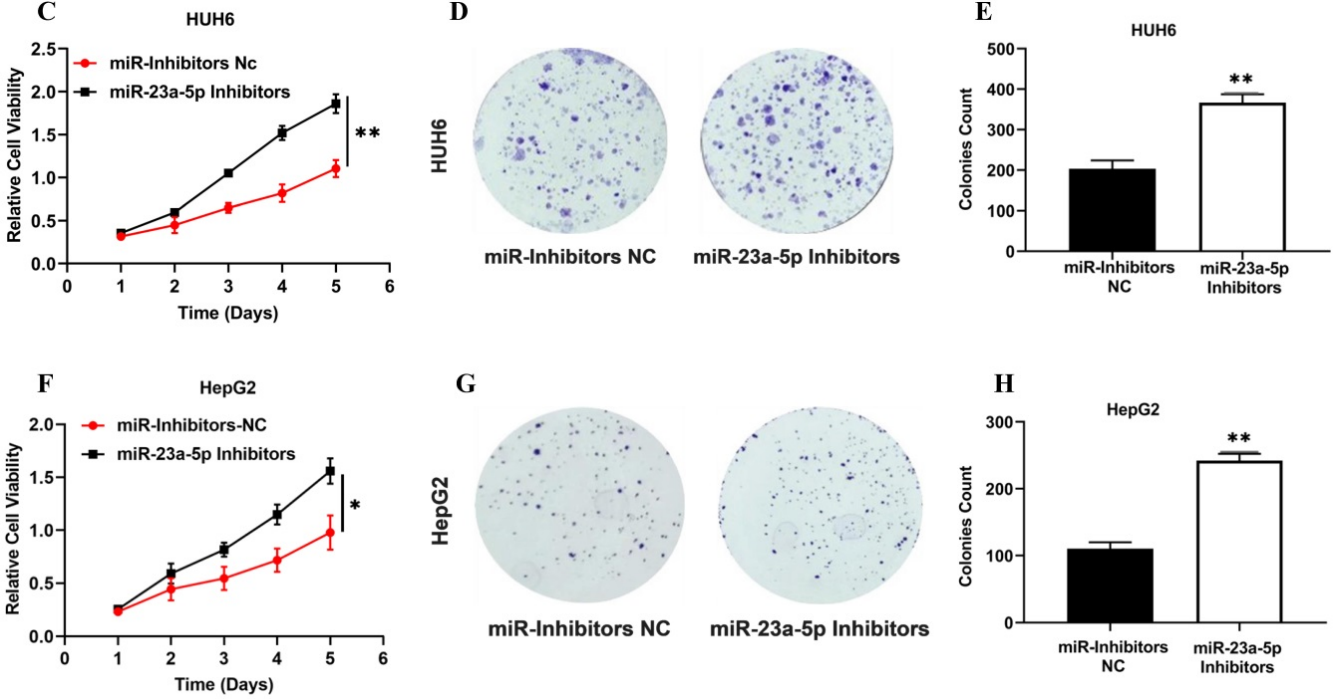

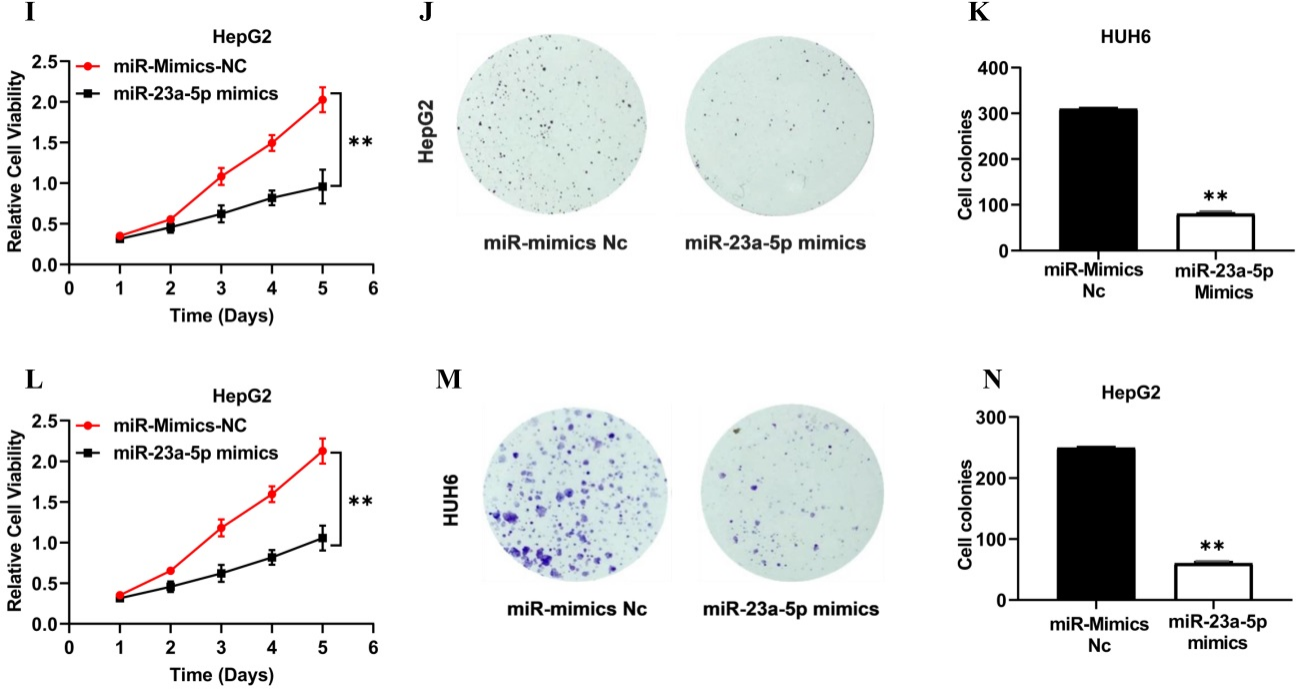
** MiR-23a-5p downregulation promotes hepatoblastoma tumorigenesis. (A)** miR-23a-5p expression in miR-23a-5p overexpressed HUH6 and HepG2 determined by qRT-PCR. **(B)** miR- 23a-5p expression in miR-23a-5p knockdown HUH6 and HepG2 cells. **(C-H)** Cell proliferation and clonogenic activity in miR-23a-5p knockdown HUH6 and HepG2 cell. **(I-N)** Cell proliferation and clonogenic activity in miR-23a-5p overexpressed HUH6 and HepG2 cells. All the data are shown as the mean ± SD. *p-value<0.05, **p<0.001, ***p<0.0001.

miR-23a-5p is a potent tumor suppressor in various cancers, including HCC & HB. However, the deregulation of miR-23a-5p resulted in the development and progression of distinct cancers. In this study, we explored the role of miR-23a-5p in hepatoblastoma. Initially, we determined the relative expression of miR-23a-5p in hepatoblastoma tissue and cell lines (HUH6 & HepG2). The qRT-PCR findings showed that miR-23a-5p a significant low expression of miR-23a-5p in primary hepatoblastoma tissue and cell lines as compared to normal hepatic tissue and (QSG7701) cells (**Figures 4A and 4B**). Next, we investigated the changes in the relative expression of miR-23a- 5p upon the subsequent knockdown and overexpression of miR-23a-5p in HUH6 and HepG2 cells. miR-23a-5p overexpression in HUH6 and HepG2 showed a considerably high expression of miR-23a-5p (**Figure 5A**). Meanwhile, miR-23a-5p knockdown in HUH6 and HepG2 results in a significant reduction in miR-23a-5p mRNA expression (**Figure 5B**). Next, we investigated the effect of miR-23a-5p knockdown and overexpression in the cell proliferation and colony formation activities in HB cells. An increased cell proliferation and colony formation activity was reported in miR-23a-5p knockdown HUH6 and HepG2 cells (**Figures 5C-H**). In the meantime, miR-23a-5p overexpressed HepG2 and HUH6 cells showed a significant reduction in cell proliferation and colony formation activity (**Figure 5I-N**). These above findings clearly suggest miR-23a-5p downregulation promotes hepatoblastoma tumorigenesis.

### miR-23a-5p interacts with Wnt3a and downregulates Wnt3a activity

**Figure 6:**
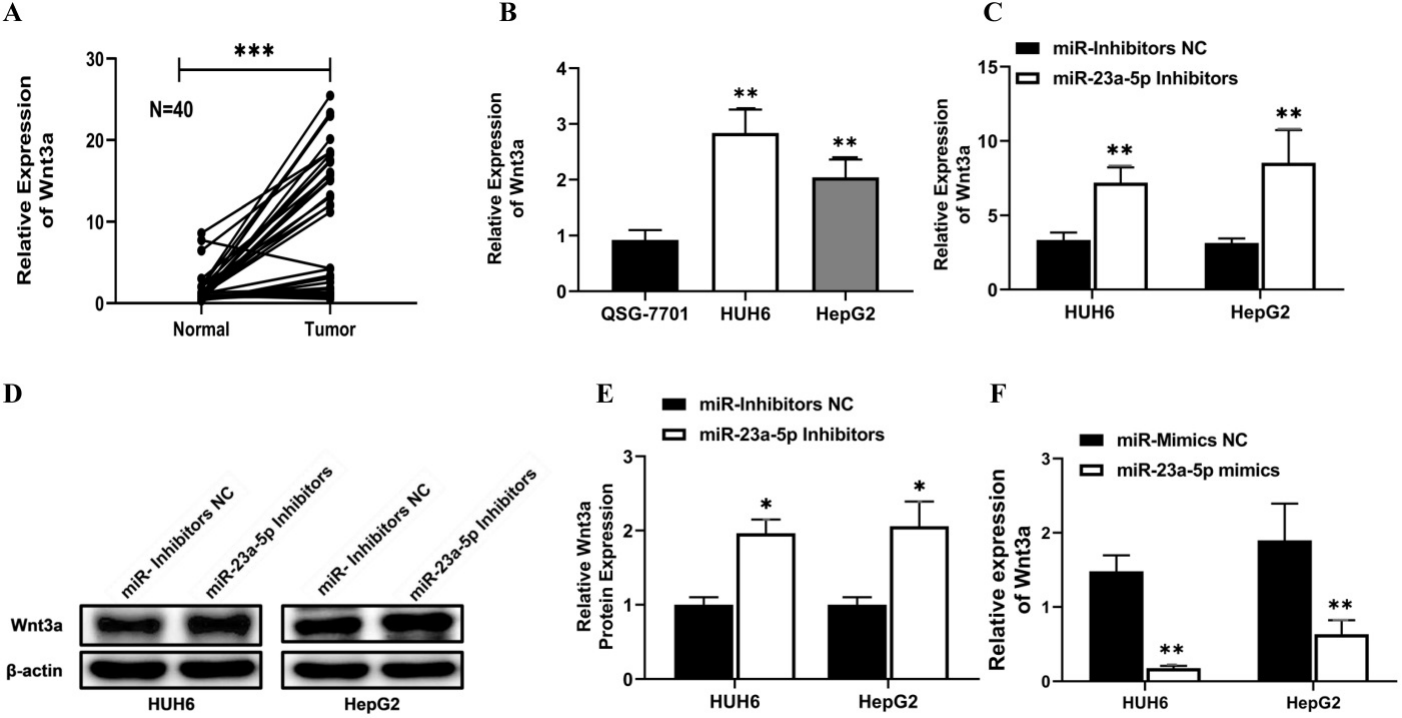

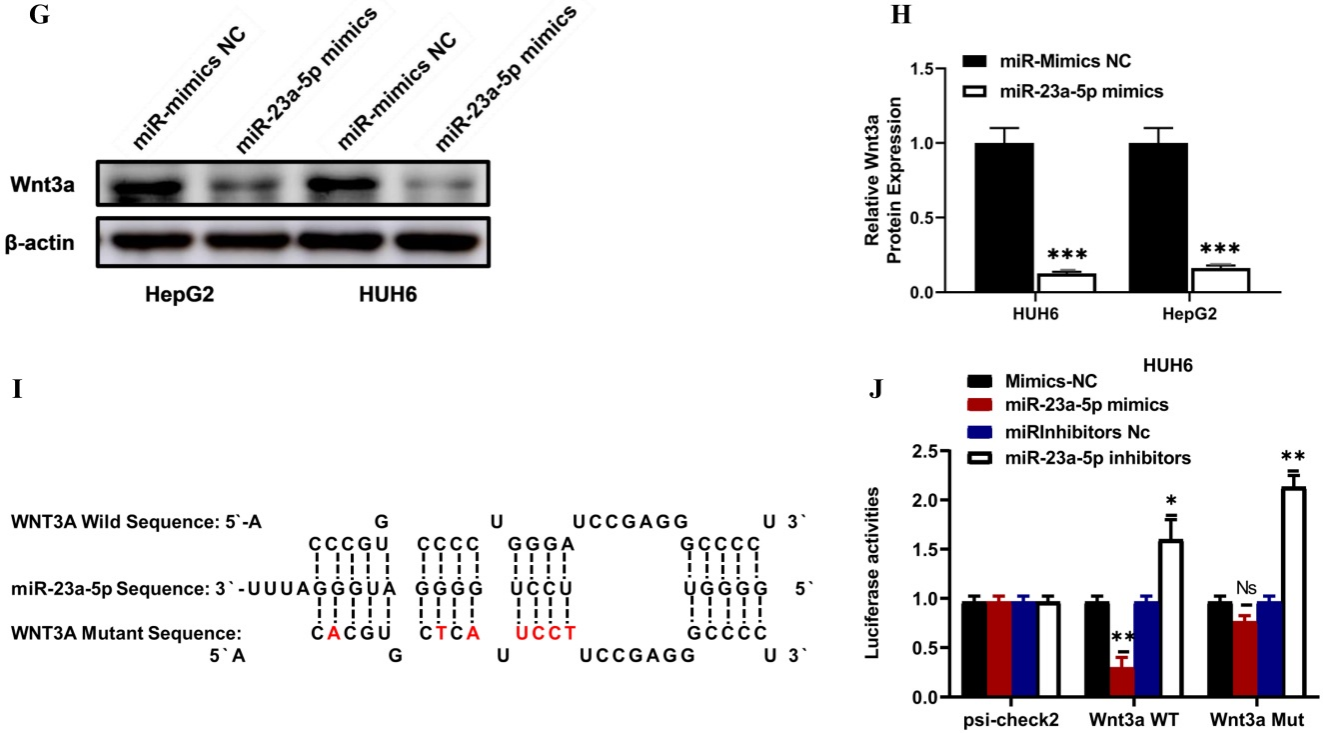

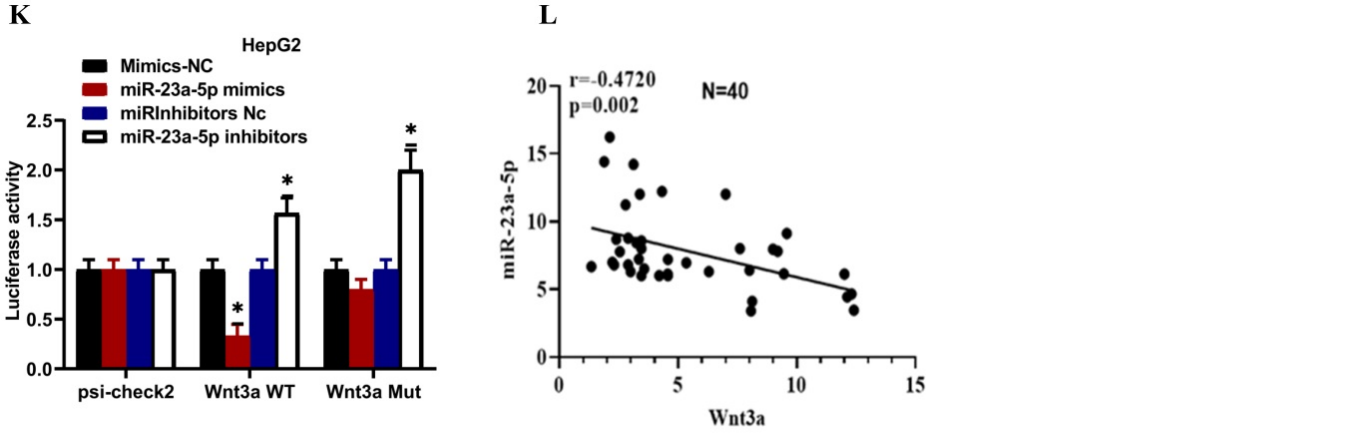
** miR-23a-5p interacts with Wnt3a and downregulates its expression. (A-B)** Wnt3a expression in hepatoblastoma tissue and cell lines was determined by qRT-PCR. **(C-E)** Wnt3a mRNA and protein expression level in miRNA 23a-5p knockdown HUH6 and HepG2 cells were determined by qRT-PCR and western blot respectively. **(F-H)** Relative Wnt3a mRNA and protein expression levels in miRNA 23a-5p overexpressed HUH6 and HepG2 cells was determined by qRT-PCR and Western blotting respectively. **(I)** The predictive binding sites of miR-23a-5p in Wnt3a predicated by using an RNA-hybrid database. **(J-K)** Interaction between miR-23a-5p and Wnt3a was confirmed by dual luciferase activities. **(L)** The Spemann's Correlation test shows a negative correlation between miR-23a-5p and Wnt3a. *p-value<0.05, **p<0.01, ***p<0.0001.

Initially, using the online bioinformatics software Target Scan, we identified Wnt3a as the target gene for miR-23a-5p. Next, we determined the relative expression of Wnt3a in hepatoblastoma primary tissue and cell lines. Wnt3a mRNA expression was found to be significantly upregulated in hepatoblastoma primary tissue and cell lines (**Figure 6A and 6B**). Conversely, miR-23a-5p expression was significantly downregulated in hepatoblastoma tissue and cell lines (**Figure 5A and 5B**). Next, we investigated the effect of miR-23a-5p knockdown and overexpression on the relative expression of Wnt3a mRNA and protein in HUH6 and HepG2 cells. A significantly high expression of Wnt3a mRNA and protein levels was observed in miR-23a-5p knockdown HUH6 and HepG2 cells (**Figure 6C and D**). Meanwhile, a significantly low Wnt3a mRNA and protein expression was reported in miR-23a-5p mimics transfected HepG2 and HUH6 cells (**Figure 6F-H**).

Next, we identified the predictive putative 3'-UTR binding sites of miR-23a-5p on Wnt3a using online bioinformatics software RNA hybrid (**Figure 6I**). Furthermore, we performed a dual luciferase assay and revealed that HUH6 and HepG2 cells co-transfected with miR-23a-3p mimics and the Wnt3a reporter vector had considerably lower luciferase activity. Meanwhile, HUH6 and HepG2 cells co-transfected with Wnt3a and with miR23a-5p inhibitors showed significantly high luciferase activity (**Figure 6J and 6K**). In addition, we performed Spearman's correlation analysis to elucidate the correlation between Wnt3a and miR23a-5p and found that their expression is negatively correlated (**Figure 6L**). These above findings indicate miR23a-5p is a tumor suppressor and downregulates Wnt3a expression in hepatoblastoma tumorigenesis.

### SNHG9 activates the Wnt/β-catenin signaling pathway via upregulation of Wnt3a

**Figure 7:**
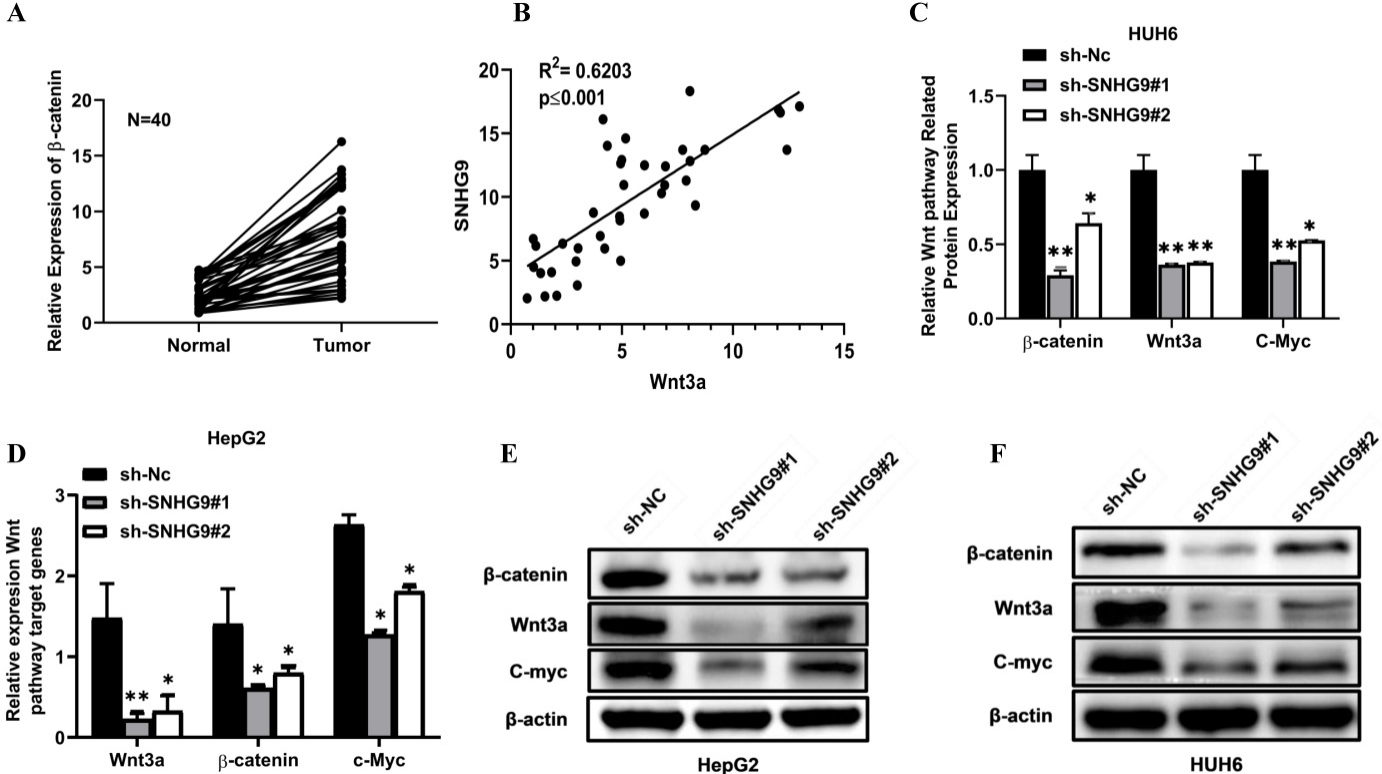

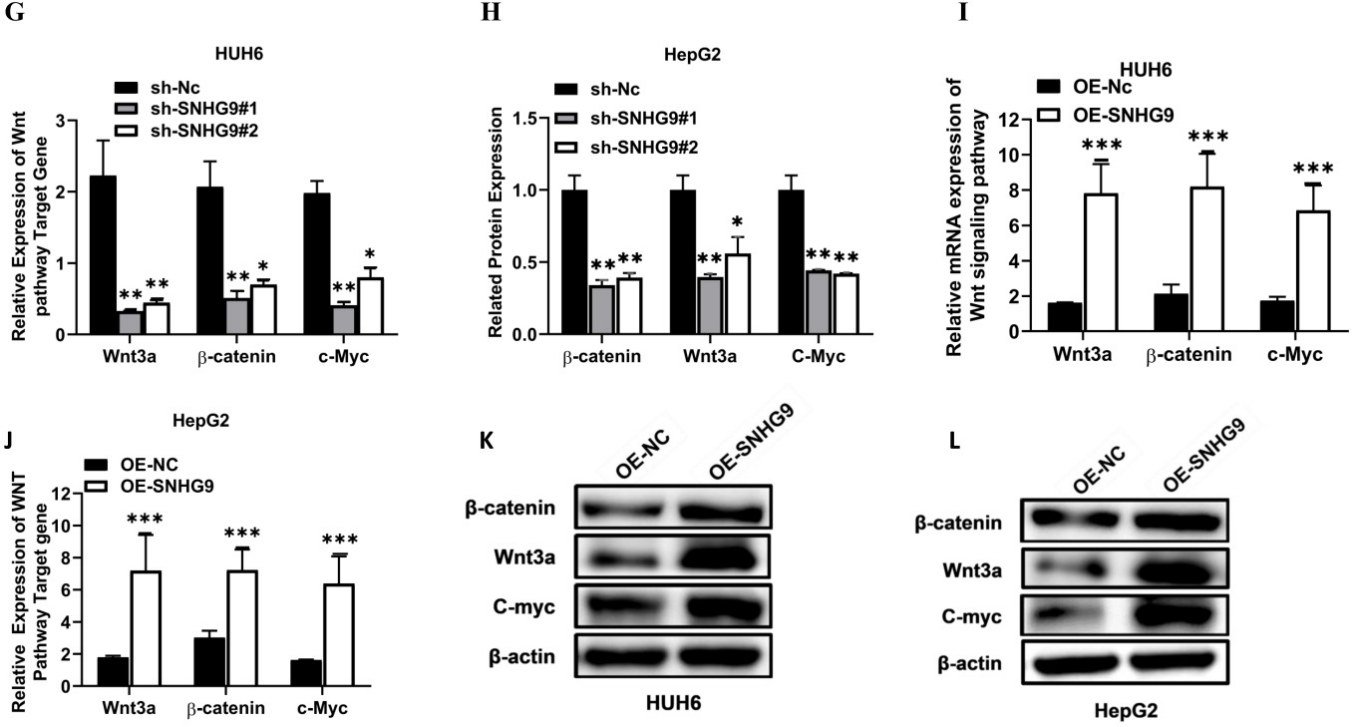

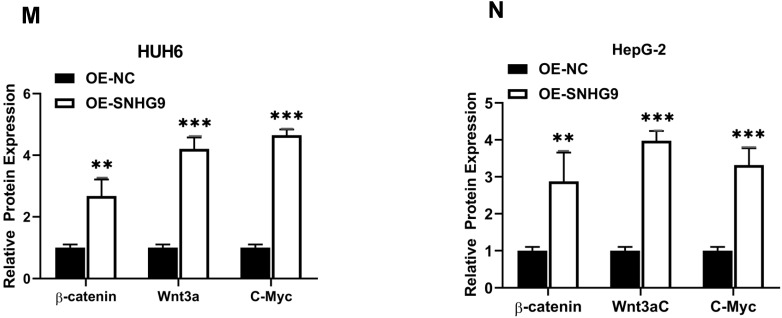
** SNHG9 activates the Wnt/ß-catenin pathway via upregulation of Wnt3a. (A)** Relative expression of β-catenin in hepatoblastoma and normal adjacent normal hepatic tissue determined by qRT-PCR. **(B)** Spearman's correlation test between SNHG9 and Wnt3a mRNA expression levels in hepatoblastoma tissue. **(C-G)** Wnt3a, ß-catenin and C-Myc mRNA expression in SNHG9 knockdown HUH6 and HepG2 determined by qRT-PCR. **(E-H)** ß-catenin, Wnt3a and C-Myc protein expression level in SNHG9 knock down HUH6 and HepG2. **(I-J)** High expression of ß-catenin, Wnt3a and C-Myc mRNA in SNHG9 overexpressed HUH6 and HepG2 determined by qRT-PCR. **(K-N)** High ß-catenin, Wnt3a and C-Myc protein expression in SNHG9 overexpressed HUH6 and HepG2 cells. *p-value<0.05, **p<0.01, ***p<0.0001.

Wnt/β-catenin signaling pathway is the most pivotal pathway and is frequently deregulated in the distinct human cancers. This study hypothesis is that SNHG9 indirectly binds to Wnt3a and activates it, eventually stimulates and activates the Wnt/β-catenin signaling pathway. To prove our hypothesis initially, we estimated the expression of β-catenin and Wnt3a in primary HB tissues, which was found to be significantly high (**Figure 7A and 6A**). Next, we performed a Pearson correlation coefficient test and a found an increased in SNHG9 expression is positively correlated with Wnt3a expression (**Figure 7B**). Next, we investigated the impact of SNHG9 depletion and overexpression on the differential expression of the distinct Wnt/β-catenin pathway associated genes and proteins, including the Wnt3a, β-catenin, c-Myc. SNHG9 silencing in HUH6 & HepG2 cell resulting in a significant reduction in Wnt3a β-catenin, and c-Myc proteins (**Figure 7C-H**). Conversely, SNHG9 was overexpressed resulting in a significant increase in the Wnt3a, ß-catenin, and C-Myc mRNA expression and related proteins (**Figure 7I-N**). These findings confirm that SNHG9 indirectly enhances the Wnt3a secretion which in turn activates the Wnt3a/β-catenin signaling pathways which in turn promotes hepatoblastoma tumorigenesis.

### miR-23a-5p and Wnt3a are involved in the activity of SNHG9 in Hepatoblastoma tumorigenesis

**Figure 8:**
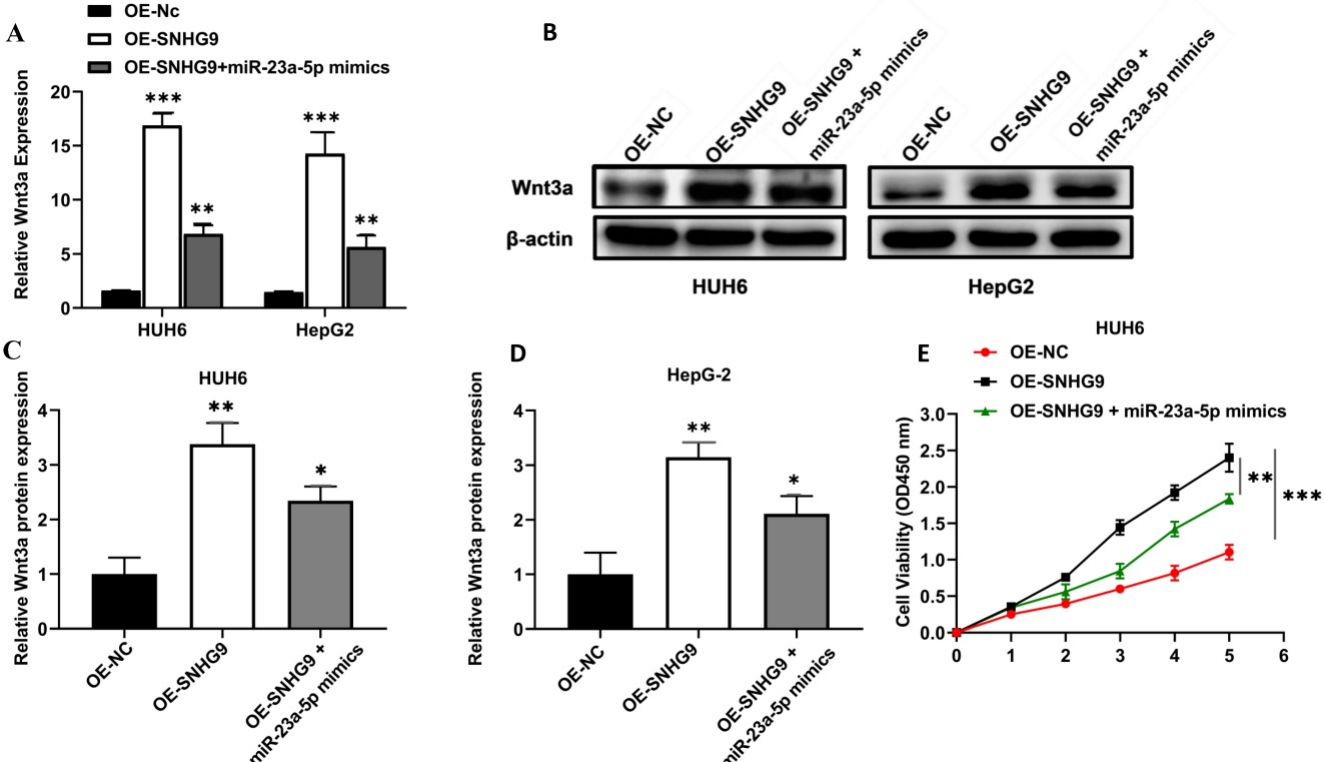

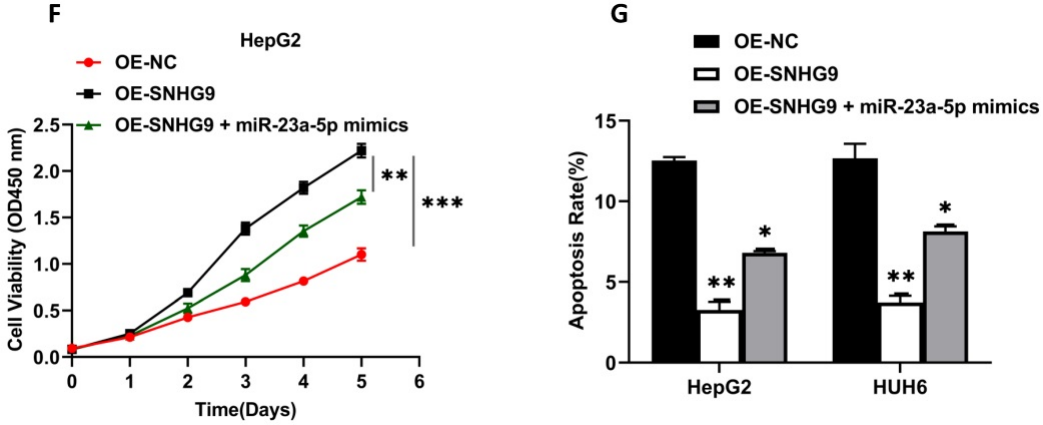
** miR-23a-5p and Wnt3a are involved in the tumorigenesis activity of SNHG9 in hepatoblastoma. (A)** Wnt3a relative expression in HUH6 and HepG2 determined by qRT-PCR. **(B)** Wnt3a protein expression in HepG2 and HuH6 determined by western blotting. **(C-D)** Quantitative estimation of relative Wnt3a protein expression in HUH6 and HepG2 cells. **(E-F)** Cell proliferation assay determined by CCK8. **(G)** Cell apoptosis activity on HB cell lines transfected with SNHG9 overexpression plasmid and miR-mimics. *p=0.05, and **p=0.01, ***p=0.0001.

Wnt3a mRNA and protein expression were significantly increased in HepG2 and HUH6 cells transfected with SNHG9 overexpression plasmids. However; a significant attenuation in Wnt3a protein and Wnt3a mRNA expression was observed in SNHG9 overexpression plasmid and miR-23a-5p mimics co-transfected HUH6 and HepG2 cells (**Figure 8A-D**). Similarly, a significantly lower cell proliferation activity was noted on SNHG9 overexpression plasmid and miR-23a-5p mimics co-transfected HUH6 and HepG2 cells than on the SNHG9 overexpressed HUH6 and HepG2 cells (**Figure 8E and 8F**). Next, cellular apoptosis was significantly increased on HB cell lines co-transfected with SNHG9 overexpression plasmid and miR-23a-5p mimics compared to SNHG9 overexpressed HepG2 and HUH6 cells (**Figure 8G**). Thus, these findings clearly suggest the involvement of has-miR-23a-5p/Wnt3a in SNHG9 activity for the progression of hepatoblastoma tumors.

### SNHG9 promotes Tumorigenesis *in-vivo*

**Figure 9:**
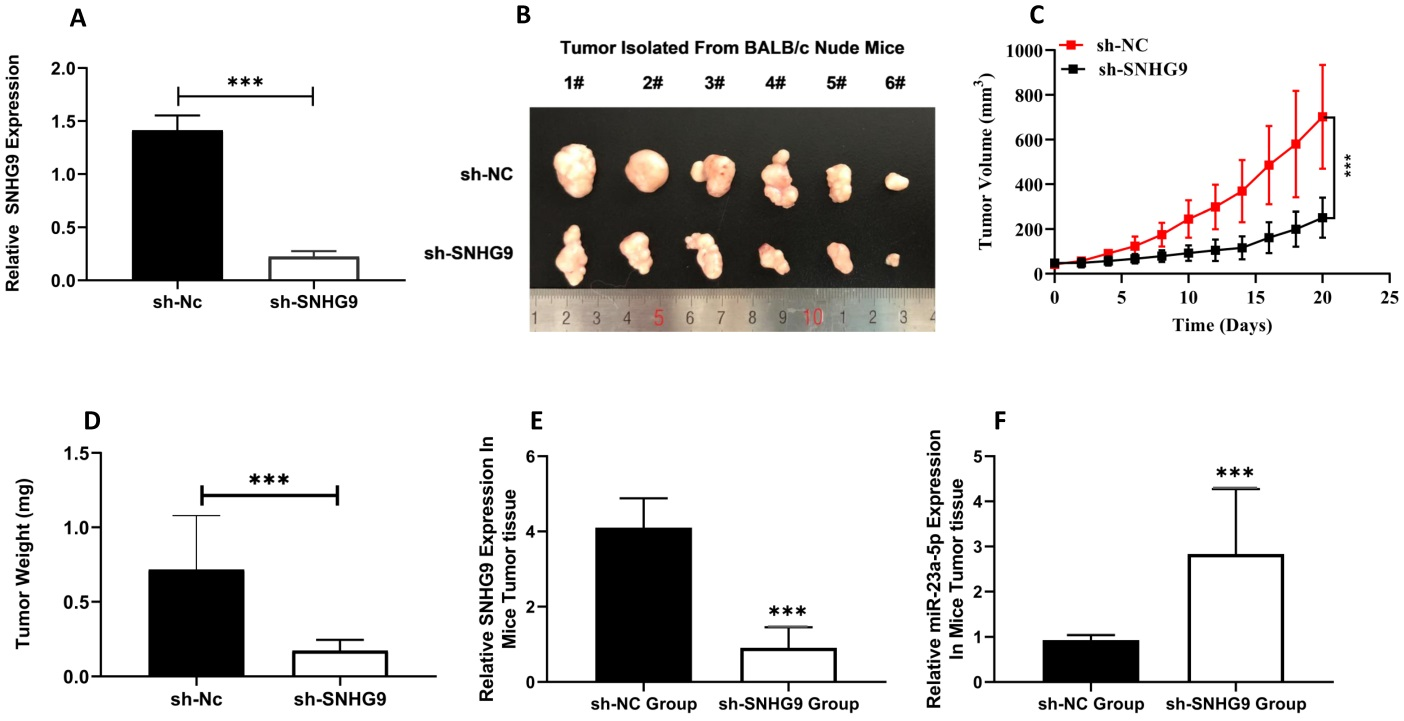

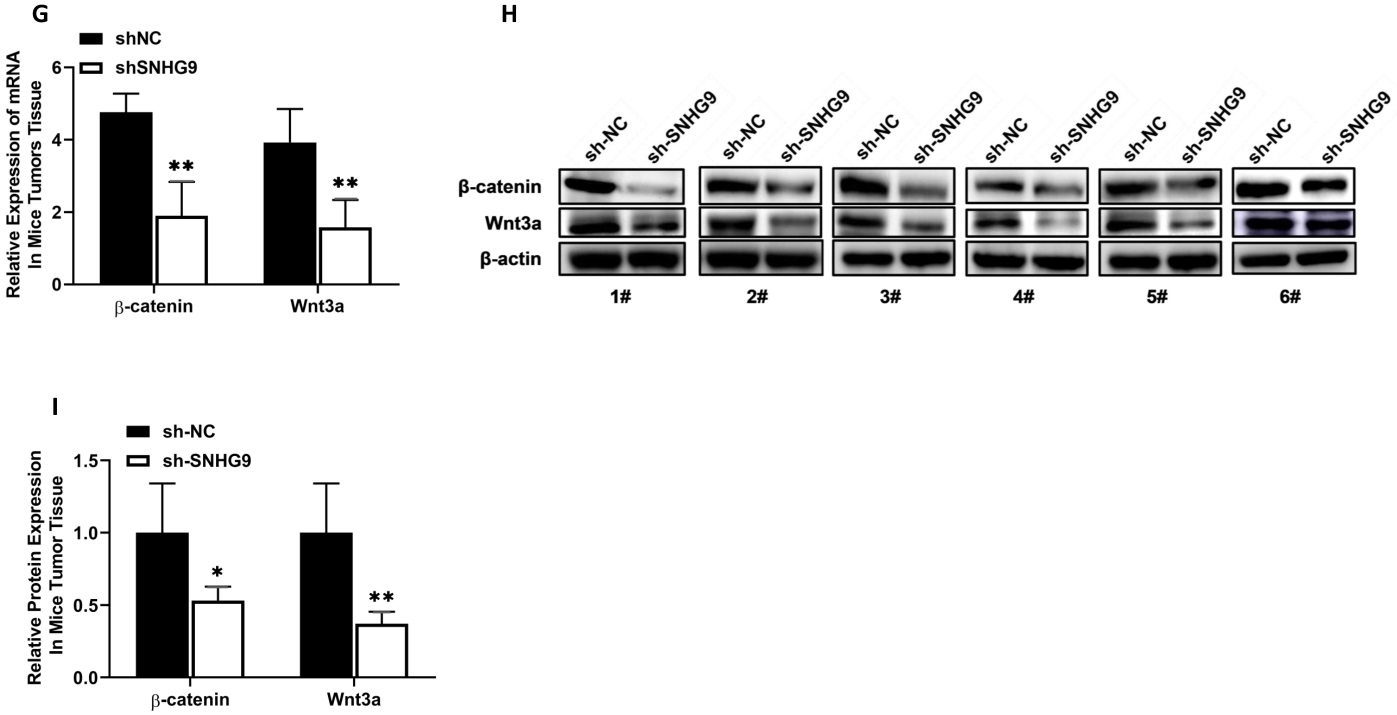
** SNHG9 Promotes Tumorigenesis *In-vivo*. (A)** SNHG9 expression in stably sh- SNHG9 or sh-NC knockdown HuH6 cells determined by qRT-PCR. **(B)** Tumors isolated from xenograft nude mice. **(C)** Average tumor volume developed in nude mice. **(D)** Average weight of tumors in nude mice measured at the end. **(E)** SNHG9 expression in tumor tissue isolated from sh- SNHG9 and sh-Nc groups of nude BALB/c mice. **(F)** miR-23a-5p expression in tumor tissue isolated from sh-SNHG9 and sh-Nc group of nude BALB/c mice. **(G)** β-catenin, Wnt3a, relative expression in tumor tissue isolated form sh-SNHG9 and sh-Nc groups of nude BALB/c mice. **(H-I)** β-catenin and Wnt3a protein expression in sh-SNHG9 tumor tissue and adjacent matched sh- NC mice tumor tissue. Data is presented as the mean ± SD. ***p-value<0.0001.

The xenograft tumorgenicity test was performed to elucidate the *in-vivo* oncogenic potential of SNHG9. Initially, the knockdown efficiency of sh-Nc and sh-SNHG9 in HUH6 was assessed, and qRT-PCR findings showed that sh-SNHG9 had a high knockdown efficiency (**Figure 9A**). To validate the *in vitro* tumorgenicity potential, stably (sh-SNHG9 or sh-NC) transfected HUH6 cells were injected subcutaneously on the posterior flank of nude mice. Twenty-four days after all the BALB/c (sh-NC group and sh-SNHG9 group) nude mice were killed by cervical dislocation, the tumors of both mice were retrieved. *In vitro*-tumorgenicity test showed a remarkably small tumor size (p<0.001) volume and weight (p<0.0001) among BALB/c nude mice injected with stably knocked down sh-SNHG9 HUH6 cells in contrast to the negative (sh-NC) control group of mice (**Figure 9B-D**). Further, we compared the relative expression of SNHG9, and miR-23a-5p and found a significant low expression of SNHG9 expression in sh-SNHG9 group mice tumor tissue than in sh-NC group mice tumor tissue (**Figure 9E**). Similarly, sh-SNHG9 group mice tumor tissue had a considerably higher miR-23a-5p was seen than sh-NC group mice tumor tissue (**Figure 9F**). Next, the relative expression of ß-catenin and Wnt3a mRNA and protein expression was also determined and we found a significantly lower β-catenin, and Wnt3a, mRNA and protein expression among the sh-SNHG9 group tumor tissue relative to the sh-NC group mice tumor tissue (**Figure 9G-I**). Collectively, based on the above experimental findings, we confirmed that SNHG9 is an oncogene and has the potential to initiate the development and progression of tumors *in vivo*.

### SNHG9 contributes to Cisplatin chemoresistance in HB cells

**Figure 10:**
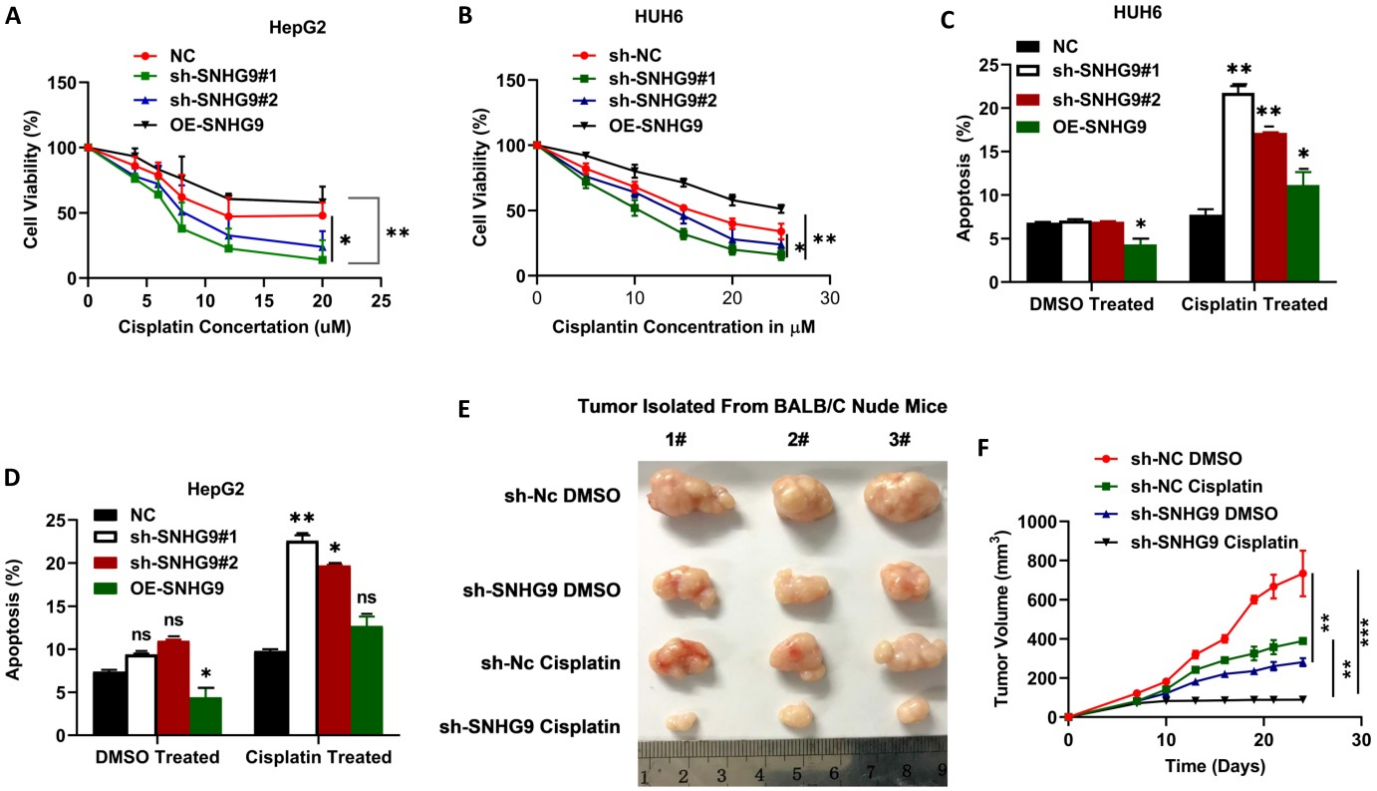

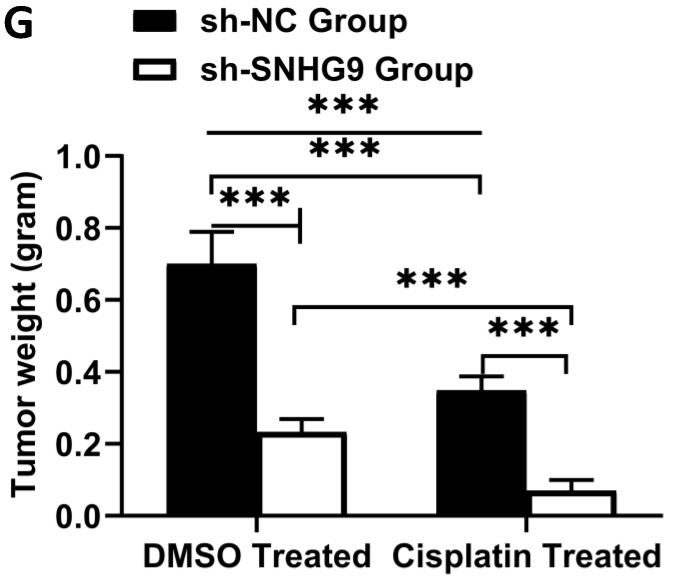
** SNHG9 contributes to cisplatin resistance in hepatoblastoma cell lines. (A-B)** Cell viability in SNHG9 knockdown/overexpressed and IC50 cisplatin treated HUH6 and HepG2 was determined by CCK8 assay. **(C-D)** Cellular apoptosis in SNHG9downregulated and upregulated along with subsequent DMSO and Cisplatin treated HepG2 and Huh6 determined by flow cytometry analysis. **(E)** Effect on the tumor growth on subsequent treatment with DMSO and cisplatin. **(F)** Tumor volume in DMSO and Cisplatin treated mice. **(G)** Tumor weight in cisplatin and DMSO treated mice. *P=0.05, **P=0.001 and ***p=0.0001.

To investigate the role of SNHG9 in cisplatin chemoresistance in HB, we initially determined the ICD (inhibitory concentration dose) of cisplatin in HUH6 and HepG2 cells and found ICD for HepG2 (12 µM) and 20 µM for HUH6. Following that, we performed CCK8 to determine the cell viability percentage after treatment with cisplatin. In comparison to the negative control group, SNHG9 siRNA knockdown HUH6 and HepG2 cells showed a substantial decrease in cell viability after the cisplatin treatment. However, SNHG9 overexpressed HUH6 and HepG2 cells showed higher cell viability even after the treatment with cisplatin drugs (**Figure 10A & 10B**). Similarly, we assessed cellular apoptosis in DMSO and Cisplatin treated cells and revealed that DMSO- treated cells had lower cellular apoptosis activity than cisplatin treated HUH6 and HepG2 cells. Meanwhile, DMSO treated SNHG9 upregulated HUH6 and HepG2 showed lower apoptosis activity compared to cisplatin treated SNHG9 overexpressed HUH6 and HepG2 cells (**Figure 10C & 10D**). As shown in (**Figure 10E-G**) significant decrease in tumor size, volume and weight in sh-SNHG9 cisplatin injected mice in comparison to sh-SNHG9 DMSO. Thus, we can confirm that SNHG9 contributes to cisplatin chemoresistance development.

## Discussion

Hepatoblastoma is the most common hepatic malignancy tumor in children with a poor prognosis 40. The disease is slowly progressive. Thus, clinicopathological examination is not adequate for the early prompt diagnosis of the disease. A significant improvement in the diagnosis, treatment and management of patients was achieved with the identification of the novel biomarker serum alpha feto protein (sAFB). However, sAFP is not highly precise and reliable in the diagnosis of tumors at an early stage. Thus, a new novel therapeutic biomarker must be identified for the early diagnosis and effective treatment of patients 41. Accumulating studies have reported that LncRNAs, a noncoding RNA (lncRNA) abnormally upregulated in distinct cancers, may have a significant role in the development and progression of distinct human cancers, including colorectal, breast, hepatocellular, pancreatic, and glioblastoma via regulating the distinct signaling pathway 42-47. Similarly, accumulating studies have shown abnormally upregulated distinct lncRNAs in hepatoblastoma. Dong R et al. on wide genomic analysis identified 2736 differently expressed lncRNAs, 1757 of which were up-regulated and 979 were downregulated in hepatoblastoma tissue 48. Previous studies have proven that SNHG9 acts as an oncogene for the development and progression of distinct human cancer including pancreatic 38, glioblastoma 39, non-small lung cancer (NSCLC) 37, bladder cancer 49, and prostrate 43. However, the biological function and underlying molecular mechanisms of SNHG9 in hepatoblastoma tumor progression are unknown and need to be elucidated.

In the current study, we elucidate the critical role of SNHG9 in hepatoblastoma progression and the underlying mechanisms. SNHG9 was conspicuously upregulated in the hepatoblastoma tissue and HB cell lines. In addition, our study findings showed that SNHG9 overexpression is closely related to advanced stage of disease and has a poor survival outcome. Next, to elucidate the biological role of SNHG9, in hepatoblastoma tumorigenesis, we knockdown and overexpressed SNHG9 in HB cell lines and performed CKK-8 and clonogenic assays. SNHG9 knock-out results in a significant depletion in cell proliferation and clonogenic activities. However, SNHG9 overexpression on HB cells enhances cell proliferation and clonogenic activity, thus confirming its involvement in the pathophysiology of hepatoblastoma tumorigenesis. We then investigated the fractional distribution of SNHG9 in cells and it was found mainly concentrated in the cytoplasm. Accumulating studies have shown that cytoplasmic lncRNAs, compete with the microRNAs by acting as ceRNAs and influence miRNA inhibitory activity on the target genes and modulating cancer progression. We assumed that SNHG9 acts as a ceRNAs in hepatoblastoma. For the confirmation using the online database, we identified the possible miRNA interacting with SNHG9 and also miR-23a-5p. The possible binding sites of miR-23a-5p on SNHG9 were identified using the RNA hybrid online bioinformatics software. The qRT-PCR finding showed reduced miR-23a-5p expression in hepatoblastoma tissue compared to normal hepatic tissue. In addition, several studies have shown tumor suppressor nature in pancreatic cancer, hepatoma, and so on 36. However, the role of miR-23a-5p in hepatoblastoma tumors is unclear. Similarly, a reduced CCK- 8 and colony formation activity was reported on HB cell lines transfected with miR-23a-5p mimics. A reduced dual luciferase activity was reported in HB cell lines co-transfected with the SNHG9 WT vector and miR-23a-5p mimics. Similarly, increased miR-23a-5p expression was reported in SNHG9 siRNA transfected HB cell lines. The Spearman's Pearson correlation analysis showed a negative correlation between SNHG9 and hsa-miR-23a-5p. Based on the above findings, we confirmed that SNHG9 directly interacts with miR-23a-5p and interferes with miRNAs activity. The canonical Wnt signaling pathway participates in normal functioning of diverse physiological processes. However, its abnormal aberrant activation results in the development and progression of cancer 17, 40, 50, 51. We identified miR23a-5p binding sites on Wnt3a using the RNA hybrid database and showed reduced luciferase activity on HB cell lines co-transfected with Wnt3aWT and miR-23a-mimics. Similarly, western blot findings and qRT-PCR findings also showed decreased Wnt3a expression in miRNA mimics transfected cells. Spearman's correlation test showed a negative correlation between Wnt3a and miR23a-5p. All these findings provide strong evidence that SNHG9 promotes hepatoblastoma tumorigenesis via downregulating miR-23a-5p and upregulation Wnt3a.

HB is a malignant tumor occurring in children. Currently, treatment of the disease mainly depends upon surgical resection, chemotherapy, and neoadjuvant chemotherapy. However, the chemotherapy response in post-operative patients progressively decreased with repeated chemotherapy, leading to failure in treatment and death of patients. In recent years, many researchers have demonstrated that lncRNAs is contributes to the development of chemoresistance. Zhang et al. (2018), reported LncRNA KcNQ1Ot1 confers cisplatin resistance in tongue cancer via miR-211-5p mediated Ezrin/Fak/Src signaling 52. Li et al reported that lncRNA UCA1 contributes in cisplatin resistance in ovarian cancer with the regulation of the miR-143/FOSL2 pathway 29. Dai et al (2020) have shown LncMALT1 regulates Cisplatin resistance in gastric cancer by the PI3K/AKT pathway 53. Similarly, Wang et al. (2021) also reported SNHG9 upregulation is associated with DDP-resistance and poor prognosis of NSCLC patients 54. However, the role of SNHG9 in cisplatin chemoresistance was not studied. In this study, we performed *in-vivo* and *in-vitro* test to demonstrate that SNHG9 contribute to cisplatin resistance in hepatoblastoma cell lines (HepG2 & HUH6). A significant reduction in cell viability was reported in IC50 cisplatin treated SNHG9 siRNA knockdown HB cell in comparison to IC50 cisplatin treated SNHG9 overexpressed HB cell lines and with DMSO treated cells. A significant reduction in tumor size, volume was reported in IC50 cisplatin and SNHG9 shRNA transfected HUH6 inoculated mice. A significant increase in cellular apoptosis among cisplatin treated HUH6 and HepG2 cells in comparison to DMSO treated HB cells. These findings confirmed that SNHG9 contributes to cisplatin resistance in HB. However, the present study has certain limitations. Due to the rarity of the diseases, the number of clinical samples taken for this study is low, which may influence the research outcomes. We performed nuclear and cytoplasmic fractional separation method to elucidate the subcellular distribution of SNHG9, however, fluorescence in-situ hybridization (FISH) was not done.

## Conclusions

In summary, to our acknowledgement, this study is the first to explore the biological function and underlying molecular mechanisms of SNHG9 in hepatoblastoma tumorigenesis. Our study findings confirmed that SNHG9 acts as an oncogene and promotes HB tumorigenesis. Eventually, we elucidate that SNHG9 promotes hepatoblastoma tumorigenesis via the miR-23a-5p/WNt3a axis. ROC analysis showed SNHG9 has high sensitivity and specificity for identification of hepatoblastoma tumors, thus it could be a potential diagnostic biomarker, and therapeutic target for the treatment and early diagnosis of HB patients.

## Supplementary Material

Supplementary table S1.

## Figures and Tables

**Table 1:** Correlation between the clinicopathological characteristics and SNHG9 expression in 40 hepatoblastoma tissue specimens

Variables	SNHG9 Expression		Χ^2^ value	p-value
	Low	High		
**Age of diagnosis**				
0-24 months	12	17	0.54	0.816
≥24 months	5	6		
**Sex**				
Male	6	5	1.338	0.247
Female	10	19		
**AFB**				
<1200 ng/ml	5	5	0.889	0.346
≥1200 ng/ml	10	20		
**AFB Final detection**				
<5 ng/ml	10	12	1.648	0.439
≥5 ng/ml	5	7		
NA	1	5		
**Histology**				
Mixed	7	6	5.765	0.056
Epithelial	1	9		
NA	9	8		
**PRETEXT**				
I-II	2	7	2.079	0.354
III-IV	10	10		
NA	4	7		
**Tumor Size**				
<500 cm^3^	4	10	3.301	0.192
≥500 cm^3^	13	13		
**Metastasis**				
YES	2	3	0.001	1
NO	14	21		
NA	0	0		

## Abbreviations

lncRNAs: Long noncoding RNAs
HB: Hepatoblastoma
sAFP: Serum Alpha Fetoproteins
OS: Overall survival
qRT-PCR: Quantitative real-time polymerase chain reaction
SDS-PAGE: Sodium Dodecylsulphate polyacrylamide gel electrophoresis
PMSF: phenylmethanesulfonylfluoride
CCK8: Cell counting kit-8
HB: Hepatoblastoma
miRNAs: MicroRNAs
ceRNAs: competitive endogenous RNA
siRNA: short interfering RNA
