# Supplementary material for: SNHG9 promotes Hepatoblastoma Tumorigenesis via miR-23a-5p/Wnt3a Axis
Source: J Cancer. 2021 Aug 22;12(20):6031–49. doi: 10.7150/jca.60748 (PMC8425203; doi:10.7150/jca.60748)
Supplement: Supplementary file 1 — Supplementary table S1. [file jcav12p6031s1.pdf]

**Supplementary Materials:**

**Additional file: Tables S1.** Primers and Oligonucleotides sequences used for qRT-PCR and cell transfection.

| Gene Name | Forward (5`-3`) | Reverse (5`-3`) |
|---|---|---|
| **SNHG9** | GCTCTCCTCTTCACTTAGGAT | ACGTGGGACAGCCAAGTTCC |
| **Wnt3a** | GCACCACCGTGGACGACAG | CCTCGCTACAGCCACCCCAC |
| **β-catenin** | CCGAATGTCTGAGGACAAGCC | GCTGCCATACCTGCTCTG GA |
| **C-Myc** | AGGGAGATCCGGAGCGAATA | TAACGTTGAGGGGCATCGTC |
| **has-miR-23a-5p** | GGGGUUCCUGGGGAUGGGAUUU | GTGCAGGGTCCGAGGT |
| **U6** | CTCGCCTTCGGCAGCACA | AACGCTTCACGAATTTGCGT |
| **GAPDH** | GTGGCTGGCTCAGAAAAAGGG | ACTGAGTGTGGCAGGGACTC |
| **Sequence of siRNA and miRNAs** | | |
| **SNHG9#1** | ACCCGAAGAGUGGCUAUAATT | UUAUAGCCACUCUUCGGGUTT |
| **SNHG9#2** | CCUCUUCACUUAGGACACUTT | AGUGUCCUAAGUGAAGAGGTT |
| **hsa-miR-23a-5p inhibitors** | AAAUCCCAUCCCCAGGAACCCC | AUCCCAUCCCCAGGAACCCCUU |
| **hsa-miR-23a-5p mimics** | GGGGUUCCUGGGGAUGGGAUUU | AUCCCAUCCCCAGGAACCCCUU |
